# Changing Trends in Cardiovascular Disease Burden in North Africa and the Middle East, 1990–2023: A Joinpoint Analysis of GBD 2023 Data

**DOI:** 10.3390/jcm15134866

**Published:** 2026-06-23

**Authors:** Hanane Ouddoud, Judah Israel Ong Lescano, Keith Pardillada Belangoy, Yoshito Nishimura, Ko Harada, Hideharu Hagiya, Quynh Thi Vu, Naohiro Iwata, Tatsuaki Takeda, Yoshito Zamami, Toshihiro Koyama

**Affiliations:** 1 Department of Health Data Science, Graduate School of Medicine, Dentistry, and Pharmaceutical Sciences, Okayama University, Okayama 7008558, Japan; pes61rgo@s.okayama-u.ac.jp (H.O.);; 2 Department of Pharmacy, School of Health Care Professions, University of San Carlos, Cebu City 6000, Philippines; 3 Division of Hematology and Oncology, Mayo Clinic, Rochester, MN 55901, USA; 4 Brookdale Department of Geriatrics and Palliative Medicine, Icahn School of Medicine at Mount Sinai, New York, NY 10029, USA; 5 Department of Infectious Diseases, Okayama University Hospital, Okayama 7008558, Japan; 6 Faculty of Pharmacy, Haiphong University of Medicine and Pharmacy, Haiphong 180000, Vietnam; 7 Department of Pharmacy, Okayama University Hospital, Okayama 7008558, Japan; 8 Department of Education and Research Center for Clinical Pharmacy, Faculty of Pharmaceutical Sciences, Okayama University, Okayama 7008558, Japan

**Keywords:** cardiovascular diseases, global burden of disease, North Africa and Middle East, risk factors, joinpoint regression

## Abstract

**Background/Objectives**: Cardiovascular disease (CVD) burden decreased in the North Africa and Middle East (NAME) region between 1990 and 2019. This study used Global Burden of Disease (GBD) 2023 data to examine whether trends in mortality, disability-adjusted life years (DALYs), incidence, and prevalence continued through 2023 across all 21 NAME countries. **Methods**: We analysed age-standardised CVD mortality, incidence, prevalence, and DALY rates from 1990 to 2023. Joinpoint regression identified changes in temporal trends and calculated the annual percent change (APC) and average annual percent change (AAPC) with 95% confidence intervals (CIs). **Results**: Age-standardised CVD mortality decreased from 579.6 per 100,000 in 1990 to 358.2 in 2023 (AAPC: −1.42%; 95% CI: −1.48 to −1.35). However, no significant reduction occurred between 2019 and 2023 (APC: −0.33%; 95% CI: −1.37 to 1.75). DALY, incidence, and prevalence rates followed similar patterns, with no significant decline in the final years of this study. Egypt was the only country with a long-term increase in CVD mortality, which accelerated after 2020 (APC: +5.20%; 95% CI: 1.20 to 12.87). High systolic blood pressure, dietary risks, lead exposure, and air pollution were the leading modifiable risk factors. **Conclusions**: The earlier decline in CVD burden in the NAME region did not clearly continue after 2019. The region is currently off track to meet Sustainable Development Goal 3.4 by 2030. Future progress may depend on improved blood pressure control, lipid management, dietary habits, and environmental risk reduction.

## 1. Introduction

Cardiovascular disease (CVD) caused 19.4 million deaths in 2021, approximately one-third of global mortality [1]. While high-income countries have achieved substantial reductions in CVD mortality over the past three decades, progress in low- and middle-income countries has been heterogeneous, with some regions experiencing stagnation or reversal [2]. The North Africa and Middle East (NAME) region, home to over 600 million people, recorded among the highest age-standardised CVD mortality rates globally in 2019, despite meaningful reductions since 1990 [2,3].

The region has undergone a rapid nutrition and epidemiological transition. Dietary patterns have shifted towards energy-dense, ultra-processed foods with high sodium and refined carbohydrate content, while urbanisation has reduced habitual physical activity [4,5]. Adult obesity prevalence now exceeds one-third in several Gulf Cooperation Council (GCC) countries, and diabetes prevalence ranks among the highest globally, particularly in Egypt and Saudi Arabia [6,7].

A previous analysis of GBD 2021 data reported declining age-standardised CVD mortality and disability-adjusted life year (DALY) rates in the NAME region between 1990 and 2021 [3]. However, GBD 2021 estimates capture only the initial phase of the COVID-19 pandemic [8]. The COVID-19 pandemic disrupted cardiovascular care and medication supply chains across the region [8,9], and fewer than 20% of adults with hypertension in the region achieve adequate blood pressure control [10].

Whether the decline in CVD mortality reported in earlier studies continued, plateaued, or reversed after 2019 remains unclear. Previous analyses described trends through to 2021 [3], but the GBD 2023 dataset now provides essential data for the post-pandemic period [4]. To address this gap, this is the first study to use GBD 2023 estimates and Joinpoint regression to evaluate post-2019 trends in CVD mortality, DALYs, incidence, and prevalence across all 21 NAME countries from 1990 to 2023. We also examined these trends by age, sex, country, CVD subtype, and attributable risk factors to provide an updated assessment of regional cardiovascular health.

## 2. Materials & Methods

### 2.1. Data Source and Study Design

This is a secondary analysis of publicly available, modelled estimates from the GBD 2023 study [4]. We examined CVD trends and risk factor attribution across 21 countries in the NAME region from 1990 to 2023. The GBD 2023 study, coordinated by the Institute for Health Metrics and Evaluation (IHME) at the University of Washington, provides standardised estimates for 375 diseases and injuries, plus 88 modifiable risk factors across 204 countries and territories [4].

The NAME region, as defined by the GBD, comprises Afghanistan, Algeria, Bahrain, Egypt, Iran, Iraq, Jordan, Kuwait, Lebanon, Libya, Morocco, Oman, Palestine, Qatar, Saudi Arabia, Sudan, Syria, Tunisia, Türkiye, United Arab Emirates, and Yemen [11]. This grouping is based on shared demographic and epidemiological transition characteristics [5]. This study was reported in accordance with the Reporting of studies Conducted using Observational Routinely-collected health Data (RECORD) statement [12], an extension of the Strengthening the Reporting of Observational Studies in Epidemiology (STROBE) guideline [13]. The completed RECORD/STROBE checklist is provided in Supplementary File S1.

### 2.2. Disease Classification

CVDs are classified as a Level 2 cause in the GBD hierarchy, comprising 12 subtypes defined by International Classification of Diseases, 10th Revision (ICD-10) codes: rheumatic heart disease (I01, I02.0, I05–I09), ischaemic heart disease (I20–I25), stroke (I60–I69, G45), hypertensive heart disease (I11), non-rheumatic valvular heart disease (I34–I37), cardiomyopathy and myocarditis (I40–I43, I51.4, B33.2), pulmonary arterial hypertension (I27.0), atrial fibrillation and flutter (I48), aortic aneurysm (I71), peripheral arterial disease (I70, I73), endocarditis (I33, I38–I39), and other cardiovascular diseases (I30, I51, I98) [4]. ICD-10 definitions and 2023 burden estimates are provided in Supplementary Table S1.

### 2.3. Epidemiological Metrics and Modelling

We extracted estimates of incidence, prevalence, mortality, years of life lost (YLLs), years lived with disability (YLDs), and DALYs for CVD and its 12 Level 2 subtypes, stratified by location, year, age, and sex. Incidence and prevalence were estimated using DisMod-MR 2.1, a Bayesian meta-regression tool. Mortality was estimated using the Cause of Death Ensemble model (CODEm), which integrates multiple statistical models and selects the best-performing ensemble based on out-of-sample predictive validity. Spatiotemporal Gaussian process regression addressed data gaps [4]. Age-standardised rates (ASR) per 100,000 population were computed using the GBD world standard population. All estimates include 95% uncertainty intervals (UIs) [4].

### 2.4. Risk Factor Assessment

Attributable CVD burden was estimated using the GBD comparative risk assessment framework for Level 3 risk factors. The estimation incorporated the concept of the theoretical minimum-risk exposure level (TMREL) to calculate population attributable fractions (PAFs). Exposure was estimated using spatiotemporal Gaussian process regression (ST-GPR); relative risks were derived from GBD meta-analyses. Risk-attributable burden was then calculated by multiplying PAFs by total CVD mortality (or DALYs), with explicit adjustment for mediation between risk factors [4].

### 2.5. Trend Analysis

Trends in ASR from 1990 to 2023 were analysed using Joinpoint Regression software (version 5.4.0.0; Statistical Research and Applications Branch, National Cancer Institute, Bethesda, MD, USA). Joinpoint fits piecewise linear models to log-transformed rates and identifies inflection points.

A grid search identified the best-fitting model, allowing for up to three joinpoints over the 33-year period. Models with 0 to 3 joinpoints were compared using the Bayesian Information Criterion (BIC), and the model with the lowest BIC was selected [14]. Statistical significance of each joinpoint was assessed using Monte Carlo permutation tests with 4499 permutations [15].

For each identified segment, the APC and its 95% CI were estimated from the slope of the log-linear model. The AAPC was calculated as the weighted average of segment-specific APCs over the full study period (1990–2023) or specified subperiods, with weights proportional to segment length. A trend was considered statistically significant if the 95% CI for the APC excluded zero (*p* < 0.05).

R software (version 4.2.2; R Foundation for Statistical Computing, Vienna, Austria) and Microsoft Excel (Microsoft Corporation, Redmond, WA, USA) were used for data processing and visualisation.

## 3. Results

### 3.1. Regional Mortality Trends

**Figure 1:**
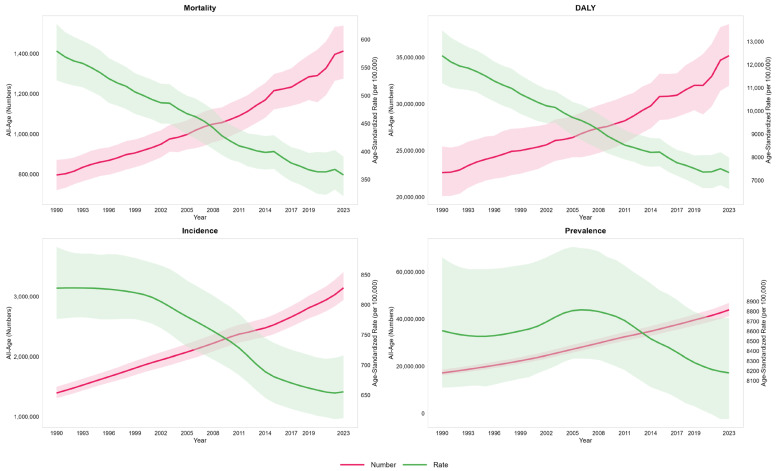
Regional trends in cardiovascular disease mortality, disability-adjusted life years (DALYs), incidence, and prevalence in the North Africa and Middle East region, 1990–2023. Pink lines indicate all-age numbers, and green lines indicate age-standardised rates per 100,000 population. Shaded areas represent 95% uncertainty intervals.

In 2023, approximately 1.41 million (95% UI: 1.28–1.54 million) CVD deaths occurred in the NAME region. The age-standardised CVD mortality rate declined from 579.6 (95% UI: 527.3–627.7) per 100,000 in 1990 to 358.2 (95% UI: 320.3–390.9) per 100,000 in 2023. The full-period AAPC from 1990 to 2023 was −1.42% (95% CI: −1.48 to −1.35). A significant decline was observed from 1990 to 2019 (AAPC: −1.54%; 95% CI: −1.61 to −1.48), followed by a period of no statistically significant change from 2019 to 2023 (APC: −0.33%; 95% CI: −1.37 to 1.75; *p* = 0.188) (Figure 1, Table 1 and Table S2; segment-specific Joinpoint fits in Figures S3 and S4).

Females had faster long-term mortality declines than males (AAPC: −1.55% vs. −1.26%). From 2019 to 2023, neither males (APC: −0.28%, 95% CI: −0.88 to 1.14) nor females (APC: −0.34%, 95% CI: −1.51 to 1.97) showed statistically significant changes (Table 1, Figure S1).

### 3.2. Country-Level Mortality Patterns

**Figure 2:**
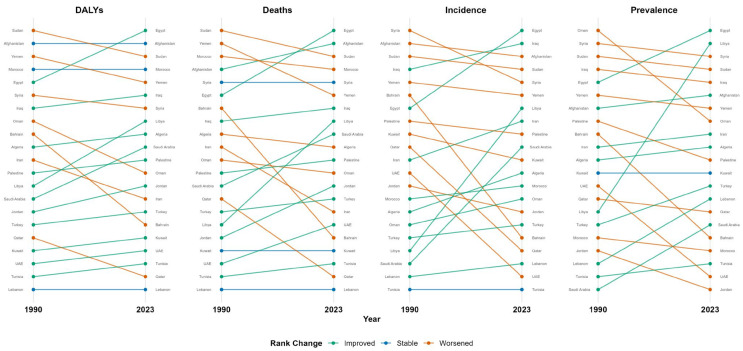
Country rankings of cardiovascular disease burden across 21 North Africa and Middle East countries, 1990 and 2023. Rankings are shown for age-standardised DALY, mortality, incidence, and prevalence rates. Lines indicate changes in rank between 1990 and 2023; green indicates improvement, orange indicates worsening, and blue indicates no change in rank. DALYs, disability-adjusted life years.

In 2023, Egypt had the highest age-standardised mortality rate (703.8 per 100,000, 95% UI: 615.2–792.8), followed by Afghanistan and Sudan, while Qatar, Lebanon, and Tunisia had the lowest rates (Figure 2 and Figure S6, Table S2). Despite having among the lowest rates, Tunisia showed a statistically significant increase in mortality during 2021–2023 (APC: +5.01%; 95% CI: 0.97 to 9.12).

Egypt was the only country with a statistically significant long-term increase in age-standardised CVD mortality (AAPC: +0.29%; 95% CI: 0.05 to 0.61). The rate of increase accelerated after 2020 (APC: +5.20%; 95% CI: 1.20 to 12.87). Qatar had the largest long-term reduction (AAPC: −3.27%, 95% CI: −3.80 to −2.76), followed by Kuwait and Bahrain (Table S2).

Among the GCC countries, no country showed statistically significant mortality changes during 2019–2023.

### 3.3. CVD Subtypes

Ischaemic heart disease (IHD) was the leading cause of CVD death throughout the study period, accounting for 52.3% of CVD deaths in 2023. IHD mortality declined from 1990 to 2023 (AAPC: −1.05%, 95% CI: −1.14 to −0.98), but showed no statistically significant change from 2020 to 2023 (APC: +0.53%, 95% CI: −0.86 to 2.61) (Table S4, Figure S10).

Stroke ranked second, accounting for 28.1% of CVD deaths in 2023, with long-term declines (AAPC: −2.39%, 95% CI: −2.48 to −2.31). Hypertensive heart disease ranked third (AAPC: −0.53%, 95% CI: −0.64 to −0.44) (Table S4). Infection-related CVDs declined: rheumatic heart disease (AAPC: −2.93%, 95% CI: −3.05 to −2.86) and pulmonary arterial hypertension (AAPC: −2.35%, 95% CI: −2.45 to −2.28).

In contrast, age-standardised mortality rates for several CVD subtypes increased over the study period. Aortic aneurysm showed the greatest increase (AAPC: +1.71%; 95% CI: 1.60 to 1.82), followed by atrial fibrillation and flutter (AAPC: +1.35%; 95% CI: 1.28 to 1.41), peripheral arterial disease (AAPC: +0.87%; 95% CI: 0.75 to 1.04), and non-rheumatic valvular heart disease (AAPC: +0.13%; 95% CI: 0.04 to 0.21) (Table S4, Figures S8 and S9).

### 3.4. Incidence and Prevalence Trends

In 2023, the NAME region recorded approximately 3.15 million (95% UI: 2.94–3.41) new CVD cases and 43.92 million (95% UI: 41.22–46.85) prevalent cases. Between 1990 and 2023, age-standardised incidence declined from 827.7 (95% UI: 776.4–896.3) to 655.4 (95% UI: 611.6–715.6) per 100,000 (AAPC: −0.71%, 95% CI: −0.73 to −0.70), as did prevalence from 8605.6 to 8180.3 per 100,000 (AAPC: −0.15%, 95% CI: −0.17 to −0.14). During 2021–2023, no statistically significant change in incidence was observed (Table 1 and Table S2).

In 2023, Egypt ranked first in both age-standardised incidence and prevalence. The UAE showed the greatest long-term decline in incidence (AAPC: −1.73%, 95% CI: −1.76 to −1.69). Statistically significant short-term increases were observed in Algeria, Tunisia, and Türkiye (2020–2023) (Figure 2, Table S2).

Age-standardised incidence was higher in males than females in 2023. Long-term declines were statistically significant for both sexes, with greater reductions in females. During 2021–2023, neither sex showed statistically significant changes in incidence (Table 1 and Table S3, Figures S1 and S2).

### 3.5. DALYs

In 2023, the NAME region recorded 35.19 million (95% UI: 31.98–38.58) DALYs attributed to CVD. Age-standardised DALY rates declined from 12,392.5 per 100,000 in 1990 to 7342.6 per 100,000 in 2023 (AAPC: −1.55%, 95% CI: −1.63 to −1.48). During 2020–2023, no statistically significant change was observed (Table 1 and Table S2).

Egypt had the highest age-standardised DALY rate in 2023 and showed a statistically significant increase during 2020–2023 (APC: +5.99%, 95% CI: 1.88 to 13.78). Qatar showed the largest long-term decline (AAPC: −3.21%, 95% CI: −3.67 to −2.82) (Table S2).

**Figure 3:**
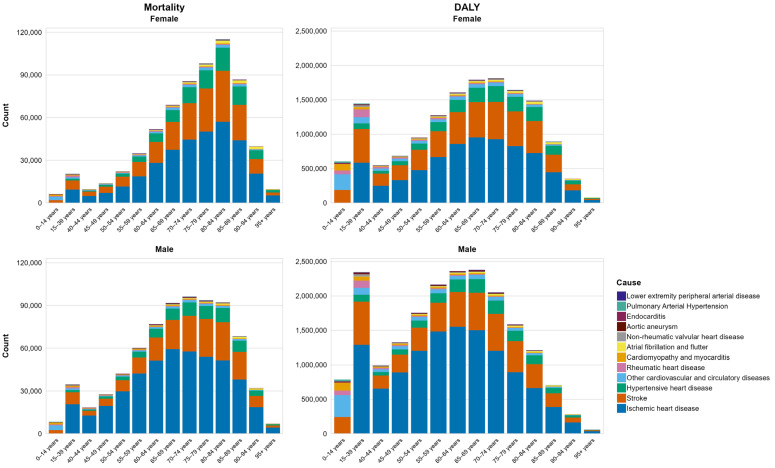
Cardiovascular disease burden by age, sex, and cause in the North Africa and Middle East region, 2023. Age- and sex-specific counts are shown for cardiovascular disease deaths and DALYs, stratified by underlying cardiovascular disease cause. Stacked bars show the contribution of ischaemic heart disease, stroke, hypertensive heart disease, rheumatic heart disease, cardiomyopathy and myocarditis, atrial fibrillation and flutter, non-rheumatic valvular heart disease, aortic aneurysm, endocarditis, pulmonary arterial hypertension, lower extremity peripheral arterial disease, and other cardiovascular and circulatory diseases. DALYs, disability-adjusted life years.

DALYs increased with age, peaking in the 65–69 years age group. Males showed higher DALY rates than females across all age groups. IHD and stroke were the leading contributors across all age categories (Figure 3).

### 3.6. Age and Sex Patterns

CVD mortality increased with age, peaking in the 80–84 years age group. Males had higher mortality counts than females across all age groups, with the sex gap widening in middle age (45–64 years). In 2023, males aged 50–69 years accounted for 31.2% of total CVD deaths despite representing 8.4% of the population (Figure 3 and Figure S7).

### 3.7. Risk Factor Attribution

**Figure 4:**
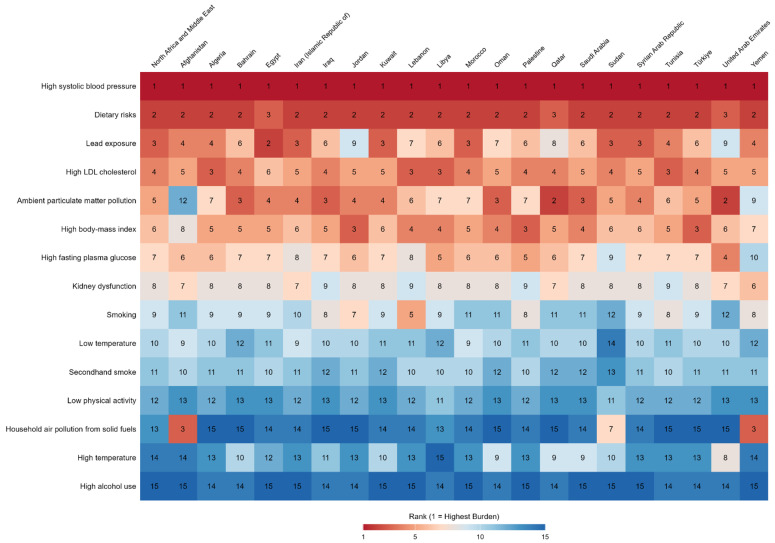
Cardiovascular mortality risk-factor rankings in the North Africa and Middle East region and its 21 countries, 2023. The heatmap ranks Level 3 risk factors by modelled attributable cardiovascular mortality burden. Rank 1 indicates the highest attributable mortality burden within each location. Lower rank numbers and warmer colours indicate higher relative contribution to cardiovascular disease mortality.

In 2023, high systolic blood pressure was the leading risk factor for CVD mortality across the NAME region, with an age-standardised death rate of 215.7 (95% UI: 175.6–255.7) per 100,000, followed by dietary risks, high LDL cholesterol, high body-mass index, and lead exposure (Figure 4).

Risk factor burdens differed by sex. Smoking-attributable DALYs were substantially higher in males than females. High BMI ranked higher among females than males in Egypt, Morocco, Saudi Arabia, and Libya.

Environmental risk factors varied across countries. In Egypt, lead exposure ranked as the third leading risk factor for CVD. Ambient particulate matter pollution contributed substantially to CVD mortality in Egypt and Iraq. Household air pollution from solid fuels was a leading driver in Afghanistan, Yemen, and Sudan. High temperature ranked among the top 10 environmental risk factors in high-income NAME countries (Figure 4 and Figure S5).

## 4. Discussion

This analysis of GBD 2023 data evaluated CVD trends across 21 countries in the NAME region from 1990 to 2023. Age-standardised CVD mortality, DALY, incidence, and prevalence rates declined between 1990 and 2019, but this progress plateaued recently. These regional metrics showed no statistically significant reduction after 2019. This plateau was observed in both sexes. At the country level, Egypt was the only country with a significant long-term increase in age-standardised CVD mortality, and this increase accelerated after 2020. IHD and stroke were the leading causes of CVD death. High systolic blood pressure, dietary risks, lead exposure, and air pollution were the leading modifiable risk factors.

Several factors may explain the plateau after 2019. First, rising metabolic risks, such as obesity, diabetes, and hypertension, continue to increase in the region. In Saudi Arabia, diabetes prevalence reached 18.7% in 2021 [6], and obesity rates in several GCC countries exceed 35% [7,16,17]. Metabolic syndrome affects 25–35% of adults in the region and doubles cardiovascular risk [17]. Second, the COVID-19 pandemic may have disrupted cardiovascular care. For instance, acute myocardial infarction presentations declined by 30% in Egypt [18]. In Lebanon, the economic crisis caused stockouts of more than 50% of essential medications [9]. Third, hypertension control rates remain low (approximately 19–20% in most countries) [19]. Together, these factors offer plausible explanations for the recent plateau, but further studies using health-system, treatment, and risk-factor data are needed to quantify their relative contributions.

Trends in GCC countries show that progress in reducing CVD mortality stopped after 2019. While these countries recorded the largest declines in the region since 1990, the recent plateau indicates that rising cardiovascular risk factors now limit the effectiveness of current prevention strategies. One factor is the demographic structure of GCC countries, where migrant workers comprise 50–90% of the population [20]. Changes in this population profile may have influenced recent trends in age-standardised mortality rates.

Egypt recorded the highest age-standardised CVD mortality rate in the region in 2023, with an acceleration in mortality trends after 2020 (APC: +5.20%; 95% CI: 1.20 to 12.87). This trend may reflect both better death registration and a real rise in disease. First, the civil registration system expanded, which increased the capture of death events, with national completeness already estimated at 96% in 2017 [21]. Second, Egypt reports high prevalence of hypertension (29.2%) and diabetes (20.9%) [6,22]. Third, lead exposure is the third-leading CVD risk factor in Egypt [23]. Sources include soil contamination, informal lead-acid battery recycling, and work in the smelting industry [23]. Improving testing and controlling these sources may help reduce the CVD burden, but the relative contribution of improved death registration versus true epidemiological change requires further investigation.

Cardiovascular disease subtypes show different trends by age and sex. Rheumatic heart disease has decreased, while conditions linked to ageing, such as atrial fibrillation and aortic aneurysm, are increasing due to population ageing and better diagnostic tools [24]. IHD mortality has plateaued after the COVID-19 pandemic [25]. Acute coronary syndromes in Middle Eastern populations occur 10–12 years earlier than in Western cohorts [26]. This pattern necessitates earlier risk screening and primary prevention strategies for younger adults.

Afghanistan, Sudan, and Yemen recorded among the highest CVD mortality rates. In these settings, health system functionality is severely compromised: in Yemen, over 50% of health facilities are non-functional [27]. GBD estimates suggest that IHD may now account for more deaths than conflict-related injuries in some of these settings [28]. Humanitarian health packages should include essential CVD medicines, including treatment for hypertension, diabetes, and secondary prevention.

The long-term AAPC of −1.42% for CVD mortality in the NAME region is lower than the rates in high-income regions (approximately −2.5% to −3.0% per year) but higher than in sub-Saharan Africa [29], reflecting an intermediate stage of epidemiological transition.

### 4.1. Clinical and Environmental Implications

The plateau in CVD trends after 2019 has direct implications for clinical practice in the NAME region. Hypertension and metabolic risks remain the main contributors to CVD mortality. Regional hypertension control rates are generally below 20% [19]. Therefore, primary care systems need to improve blood pressure and lipid management [30]. Protocols like the WHO HEARTS package provide a practical framework for better clinical control [31]. Because lead exposure is a major CVD risk factor in Egypt, physicians treating patients with resistant hypertension should screen for environmental exposures [32]. Current trends show the region is off track for the Sustainable Development Goal 3.4 target by 2030 [33]. Reaching this target depends on both clinical care and environmental risk reduction.

### 4.2. Limitations

This study has several limitations. First, conclusions about the post-2019 plateau are based on a short period of approximately 3–4 years that overlaps with the COVID-19 pandemic. Estimates for the most recent years may be affected by reporting delays, healthcare disruptions, and cause-of-death misclassification; therefore, trend changes during 2020–2023 should be interpreted cautiously and may be revised in future GBD releases as additional data become available. Second, GBD estimates rely on statistical modelling to address data gaps, and data quality and completeness vary across the NAME region. In countries affected by conflict or health-system disruption, including Afghanistan, Sudan, Syria, and Yemen, vital registration, diagnostic capacity, and cause-of-death certification may be incomplete. Third, country-level differences and short-term trend changes should be interpreted considering their uncertainty intervals rather than point estimates alone. Fourth, risk-factor rankings are modelled estimates derived from the GBD comparative risk assessment framework and should not be interpreted as direct causal effects measured in this study. Fifth, as an ecological analysis, this study describes population-level trends and does not establish individual-level causality.

## 5. Conclusions

CVD mortality, DALY, incidence, and prevalence rates declined in the NAME region between 1990 and 2019, but this progress plateaued. These regional metrics showed no significant reduction after 2019. Egypt is the only country with a long-term increase in CVD mortality, which accelerated after 2020. These findings indicate that the current rate of reduction is insufficient to address rising metabolic and environmental risks. Reducing the CVD burden requires health systems to focus on primary prevention, specifically hypertension control, dietary habits, tobacco reduction, and environmental health, as well as acute care. Without these measures, the NAME region will not reach the SDG 3.4 target by 2030.

## Figures and Tables

**Table 1:** Cardiovascular disease burden by sex in the NAME region, 1990–2023.

Measure	Sex	Absolute Number in Millions (2023)	ASR (Per 100,000), 2023	AAPC (1990–2023)
Incidence	Both	3.15 (2.94–3.41)	655.45 (611.57–715.56)	−0.71 (−0.73, −0.70) *
	Male	1.75 (1.62–1.90)	729.80 (679.98–791.75)	−0.57 (−0.59, −0.56) *
	Female	1.40 (1.31–1.52)	584.89 (546.49–632.99)	−0.85 (−0.86, −0.84) *
Prevalence	Both	43.92 (41.22–46.85)	8180.33 (7715.09–8733.38)	−0.15 (−0.17, −0.14) *
	Male	24.98 (23.37–26.82)	9053.98 (8496.36–9712.01)	−0.12 (−0.13, −0.11) *
	Female	18.93 (17.83–20.23)	7270.45 (6866.73–7765.35)	−0.20 (−0.22, −0.18) *
Deaths	Both	1.41 (1.28–1.54)	358.19 (320.28–390.88)	−1.42 (−1.48, −1.35) *
	Male	0.75 (0.65–0.85)	388.08 (336.45–437.24)	−1.26 (−1.33, −1.21) *
	Female	0.66 (0.58–0.75)	331.16 (289.01–375.58)	−1.55 (−1.63, −1.46) *
DALYs	Both	35.19 (31.98–38.58)	7342.57 (6634.34–7991.68)	−1.55 (−1.63, −1.48) *
	Male	20.01 (17.45–22.93)	8180.97 (7165.73–9237.07)	−1.42 (−1.54, −1.33) *
	Female	15.18 (13.33–17.24)	6486.21 (5706.17–7290.98)	−1.68 (−1.77, −1.60) *

* AAPC was considered statistically significant if *p* < 0.05 and the 95% CI did not include zero. AAPC = average annual percent change; ASR = age-standardised rate; DALY = disability-adjusted life years.

## Data Availability

The datasets supporting the conclusions of this article are available in the IHME Global Burden of Disease Results Tool repository, https://vizhub.healthdata.org/gbd-results/ (accessed on 18 November 2025). The full set of country-specific Joinpoint regression results, source CSV files, and analysis scripts (R 4.2.2; Joinpoint v5.4.0.0) used to generate the figures and tables in this article are archived on Zenodo https://doi.org/10.5281/zenodo.19909178.

## Supplementary Materials

The following supporting information can be downloaded at: https://www.mdpi.com/article/10.3390/jcm15134866/s1, Table S1: ICD-10 definitions and 2023 burden estimates for cardiovascular disease subtypes in the NAME region; Table S2: Country-specific trends in age-standardised rates of cardiovascular deaths, DALYs, YLLs, YLDs, incidence, and prevalence in NAME countries, 1990–2023; Table S3: Joinpoint regression results for cardiovascular disease incidence, mortality, and DALYs in the NAME region by sex, 1990–2023; Table S4: Regional trends in age-standardised rates of cardiovascular burden stratified by specific cause, both sexes, 1990–2023; Figure S1: Time trends in age-standardised rates and all-age numbers of mortality, DALYs, incidence, and prevalence due to cardiovascular diseases in the NAME region, females, 1990–2023; Figure S2: Time trends in age-standardised rates and all-age numbers of mortality, DALYs, incidence, and prevalence due to cardiovascular diseases in the NAME region, males, 1990–2023; Figure S3: Joinpoint regression segments for cardiovascular disease age-standardised mortality, DALYs, incidence, and prevalence, NAME region, both sexes, 1990–2023; Figure S4: Joinpoint regression segments for cardiovascular disease age-standardised mortality and DALYs by sex, NAME region, 1990–2023; Figure S5: Comparison of cardiovascular disease risk factors across NAME countries in terms of DALYs, both sexes, 2023; Figure S6: Geographical distribution of age-standardised cardiovascular mortality and DALY rates in the NAME region, 1990 versus 2023; Figure S7: Age-standardised mortality and DALY rates for cardiovascular diseases by age group and sex in the NAME region, 1990 versus 2023; Figure S8: Heatmap of average annual percentage change in DALYs for cardiovascular disease causes across NAME countries, 1990–2023; Figure S9: Heatmap of average annual percentage change in deaths for cardiovascular disease causes across NAME countries, 1990–2023; Figure S10: Comparison of NAME countries by age-standardised mortality and DALY rates attributable to specific cardiovascular disease causes, 2023; Supplementary Checklist S1: RECORD/STROBE Checklist.
