# Supplementary material for: Changing Trends in Cardiovascular Disease Burden in North Africa and the Middle East, 1990–2023: A Joinpoint Analysis of GBD 2023 Data
Source: J Clin Med. 2026 Jun 23;15(13):4866. doi: 10.3390/jcm15134866 (PMC13360749; doi:10.3390/jcm15134866)
Supplement: Supplementary file 1 [file jcm-15-04866-s001.zip › jcm-4342510-supplementary.pdf]

Supplementary Material

Table of Contents

Supplementary Tables

Table S1. ICD-10 definitions and 2023 burden estimates for cardiovascular disease subtypes in the NAME region.
Table S2. Country-specific trends in age-standardised rates of cardiovascular deaths, DALYs, YLLs, YLDs, incidence, and prevalence in NAME countries, 1990–2023.
Table S3. Joinpoint regression results for cardiovascular disease incidence, mortality, and DALYs in the NAME region by sex, 1990–2023.
Table S4. Regional trends in age-standardised rates of cardiovascular burden stratified by specific cause, both sexes, 1990–2023.

Supplementary Figures

Figure S1. Time trends in age-standardised rates and all-age numbers of mortality, DALYs, incidence, and prevalence due to cardiovascular diseases in the NAME region, females, 1990–2023.
Figure S2. Time trends in age-standardised rates and all-age numbers of mortality, DALYs, incidence, and prevalence due to cardiovascular diseases in the NAME region, males, 1990–2023.
Figure S3. Joinpoint regression segments for cardiovascular disease age-standardised rates in the NAME region, both sexes, 1990–2023.
Figure S4. Joinpoint regression segments for cardiovascular disease age-standardised mortality and DALYs by sex, NAME region, 1990–2023.
Figure S5. Comparison of CVD risk factors across NAME countries in terms of DALYs, both sexes, 2023.
Figure S6. Geographical distribution of age-standardised cardiovascular mortality and DALY rates in the NAME region, 1990 versus 2023.
Figure S7. Age-standardised mortality and DALY rates for cardiovascular diseases by age group and sex in the NAME region, 1990 versus 2023.
Figure S8. Heatmap of AAPC in DALYs for cardiovascular disease causes across NAME countries, 1990–2023.
Figure S9. Heatmap of average annual percentage change (AAPC) in deaths for cardiovascular disease causes across NAME countries, 1990–2023.
Figure S10. Comparison of NAME countries by age-standardised mortality and DALY rates attributable to specific cardiovascular disease causes, 2023.

Supplementary Checklist

Checklist S1. The RECORD statement – checklist of items, extended from the STROBE statement, that should be reported in observational studies using routinely collected health data.

Table S1. ICD-10 definitions and 2023 burden estimates for cardiovascular disease subtypes in the NAME region.

CVD subtype	ICD-10 code	2023 deaths number (95% UI)	2023 DALYs number (95% UI)	2023 incidence number (95% UI)	2023 prevalence number (95% UI)
**Cardiovascular diseases**	All codes listed below	1,412,959 (1,275,878-1,539,067)	35,190,299 (31,976,241-38,578,393)	3,146,186 (2,944,722-3,410,831)	43,916,746 (41,217,031-46,848,808)
**Rheumatic heart disease**	I01, I02.0, I05-I09	10,835 (7,202-15,456)	587,545 (425,626-812,269)	218,697 (174,708-265,487)	3,358,796 (2,710,270-4,048,430)
**Ischaemic heart disease**	I20-I25	798,317 (711,742-896,616)	19,191,847 (17,044,628-21,497,376)	1,381,219 (1,191,287-1,583,092)	21,224,054 (19,083,032-23,688,024)
**Stroke**	I60-I69, G45	377,930 (308,912-455,659)	9,329,221 (7,689,175-11,369,216)	642,385 (585,486-704,877)	6,518,415 (6,241,002-6,819,434)
**Hypertensive heart disease**	I11	150,156 (117,410-193,965)	3,062,038 (2,376,509-3,947,511)	NA	1,081,515 (857,515-1,347,025)
**Non-rheumatic valvular heart disease**	I34-I37	7,779 (5,552-10,624)	227,349 (162,449-304,609)	50,979 (45,422-56,952)	557,576 (485,158-638,661)
**Cardiomyopathy and myocarditis**	I40-I43, I51.4, B33.2	12,286 (8,293-19,080)	514,174 (335,753-785,029)	64,460 (49,879-83,402)	252,722 (204,939-306,560)
**Pulmonary arterial hypertension**	I27.0	1,850 (1,156-2,930)	87,493 (52,086-141,417)	3,027 (2,420-3,681)	12,113 (9,858-15,071)
**Atrial fibrillation and flutter**	I48	12,916 (10,145-15,852)	324,272 (255,317-411,175)	186,276 (140,995-240,118)	1,829,952 (1,348,312-2,322,959)
**Aortic aneurysm**	I71	4,149 (3,380-4,928)	109,074 (88,696-131,120)	NA	NA
**Peripheral arterial disease**	I70, I73	1,285 (1,062-1,556)	47,693 (33,872-68,801)	525,521 (396,436-680,330)	4,563,365 (3,448,501-5,966,454)
**Endocarditis**	I33, I38-I39	2,833 (2,239-3,725)	108,898 (80,375-149,832)	73,622 (63,341-83,486)	16,098 (14,102-18,534)
**Other cardiovascular and circulatory diseases**	I30, I51, I98	32,622 (24,592-42,149)	1,600,695 (1,227,249-1,983,133)	NA	8,629,525 (6,886,404-10,737,539)

Table S2. Country-specific trends in age-standardised rates of cardiovascular deaths, DALYs, YLLs, YLDs, incidence, and prevalence in NAME countries, 1990–2023.

Location	Measure	ASR 1990 (95% UI)	ASR 2023 (95% UI)	AAPC (95% CI)	Recent APC (95% CI)	Recent Period
**North Africa and Middle East**						
	Deaths	579.6 (527.3–627.7)	358.2 (320.3–390.9)	−1.42 (−1.48–−1.35) *	−0.33 (−1.37–1.75)	2019–2023
	DALYs	12392.5 (11195.6–13488.1)	7342.6 (6634.3–7991.7)	−1.55 (−1.63–−1.48) *	0.21 (−0.90–2.18)	2020–2023
	YLLs	11860.3 (10695.3–13029.0)	6849.4 (6168.2–7483.2)	−1.62 (−1.71–−1.55) *	0.25 (−0.93–2.34)	2020–2023
	YLDs	532.2 (398.7–695.2)	493.2 (370.4–652.7)	−0.23 (−0.24–−0.22) *	−0.07 (−0.32–0.10)	2021–2023
	Incidence	827.7 (776.4–896.3)	655.4 (611.6–715.6)	−0.71 (−0.73–−0.70) *	0.06 (−0.38–0.34)	2021–2023
	Prevalence	8605.6 (8031.1–9346.4)	8180.3 (7715.1–8733.4)	−0.15 (−0.17–−0.14) *	−0.24 (−0.47–0.07)	2020–2023
**AFGHANISTAN**						
	Deaths	786.6 (715.1–856.3)	587.0 (527.2–658.5)	−0.89 (−1.01–−0.78) *	−0.89 (−1.01–−0.78) *	1990–2023
	DALYs	16775.0 (14932.0–18336.5)	11908.9 (10555.9–13418.6)	−1.06 (−1.17–−0.95) *	−1.06 (−1.17–−0.95) *	1990–2023
	YLLs	16238.1 (14433.4–17800.1)	11379.5 (10052.2–12832.0)	−1.10 (−1.22–−0.99) *	−1.10 (−1.22–−0.99) *	1990–2023
	YLDs	536.9 (404.2–689.1)	529.4 (396.2–685.7)	−0.04 (−0.06–−0.03) *	0.39 (0.18–0.61) *	2021–2023
	Incidence	970.1 (889.2–1078.8)	794.1 (739.2–859.0)	−0.61 (−0.63–−0.59) *	0.17 (0.00–0.40)	2019–2023
	Prevalence	8907.3 (8092.1–9850.3)	8816.4 (8262.0–9599.5)	−0.03 (−0.04–−0.02) *	−0.21 (−0.33–−0.04) *	2014–2023
**ALGERIA**						
	Deaths	531.8 (467.5–599.4)	322.6 (263.7–391.7)	−1.49 (−1.55–−1.45) *	−0.95 (−2.38–−0.04) *	2021–2023
	DALYs	11253.9 (9867.0–12805.1)	6843.1 (5719.6–8277.8)	−1.49 (−1.55–−1.44) *	−0.11 (−1.26–0.91)	2021–2023
	YLLs	10728.8 (9371.9–12176.6)	6346.2 (5233.7–7786.0)	−1.57 (−1.64–−1.51) *	−0.11 (−1.40–1.00)	2021–2023
	YLDs	525.1 (391.9–681.7)	496.9 (372.9–654.5)	−0.17 (−0.18–−0.16) *	−0.01 (−0.25–0.13)	2021–2023
	Incidence	797.0 (739.7–869.8)	608.4 (566.6–668.0)	−0.81 (−0.82–−0.80) *	0.45 (0.29–0.78) *	2020–2023
	Prevalence	8584.8 (8018.1–9356.7)	8217.1 (7593.9–8896.6)	−0.14 (−0.14–−0.13) *	−0.15 (−0.33–−0.03) *	2014–2023
**BAHRAIN**						
	Deaths	591.4 (541.1–646.3)	202.5 (169.2–233.4)	−3.17 (−3.43–−2.99) *	−0.62 (−3.79–4.21)	2020–2023
	DALYs	11457.0 (10473.5–12417.7)	3929.0 (3361.7–4465.1)	−3.17 (−3.33–−3.01) *	0.62 (−2.01–4.97)	2020–2023
	YLLs	10940.3 (9977.5–11898.8)	3514.7 (2983.8–4024.7)	−3.36 (−3.52–−3.18) *	0.70 (−2.19–5.36)	2020–2023
	YLDs	516.7 (389.3–663.6)	414.3 (310.0–535.4)	−0.67 (−0.69–−0.65) *	−0.25 (−0.56–−0.02) *	2021–2023
	Incidence	911.9 (847.6–985.4)	518.8 (479.4–568.3)	−1.70 (−1.73–−1.66) *	0.34 (−0.08–1.00)	2019–2023
	Prevalence	8766.5 (8008.9–9625.4)	6830.3 (6300.8–7385.7)	−0.76 (−0.77–−0.74) *	−0.12 (−0.44–0.07)	2021–2023
**EGYPT**						
	Deaths	662.4 (590.1–737.0)	703.8 (615.2–792.8)	0.29 (0.05–0.61) *	5.20 (1.20–12.87) *	2020–2023
	DALYs	14873.3 (13240.7–16501.4)	13879.5 (12107.0–15442.7)	−0.09 (−0.33–0.16)	5.99 (1.88–13.78) *	2020–2023
	YLLs	14279.5 (12613.0–15855.4)	13307.0 (11544.3–14861.2)	−0.02 (−0.30–0.22)	5.53 (0.29–14.01) *	2020–2023
	YLDs	593.8 (444.3–773.3)	572.5 (433.4–749.1)	−0.11 (−0.13–−0.10) *	0.00 (−0.23–0.22)	2021–2023
	Incidence	902.2 (835.5–974.3)	823.6 (758.8–891.8)	−0.28 (−0.32–−0.25) *	−0.36 (−1.36–0.14)	2021–2023
	Prevalence	9419.3 (8783.2–10231.3)	9572.5 (8938.8–10277.4)	0.05 (0.03–0.07) *	−0.24 (−0.60–0.30)	2020–2023
**IRAN**						
	Deaths	530.8 (468.3–587.2)	217.5 (179.8–250.6)	−2.67 (−2.89–−2.49) *	−3.65 (−8.62–−0.13) *	2016–2023
	DALYs	10689.0 (9308.3–11961.3)	4211.9 (3589.8–4770.2)	−2.76 (−2.96–−2.65) *	−3.64 (−8.27–−2.98) *	2016–2023
	YLLs	10161.1 (8758.8–11440.0)	3705.1 (3131.3–4241.9)	−3.01 (−3.25–−2.89) *	−4.03 (−9.30–−3.24) *	2016–2023
	YLDs	527.9 (392.0–682.7)	506.9 (372.0–671.6)	−0.11 (−0.14–−0.07) *	−0.49 (−0.76–−0.20) *	2015–2023
	Incidence	845.0 (783.0–927.0)	650.5 (591.5–720.5)	−0.80 (−0.84–−0.78) *	−0.81 (−1.61–−0.26) *	2020–2023
	Prevalence	8676.9 (7990.8–9616.7)	8465.1 (7831.6–9296.6)	−0.05 (−0.08–−0.00) *	−0.45 (−0.65–−0.22)	2015–2023
**IRAQ**						
	Deaths	544.8 (471.9–603.2)	429.7 (362.1–490.4)	−0.72 (−0.80–−0.65) *	1.40 (−0.51–2.71)	2021–2023
	DALYs	12020.0 (10340.1–13457.2)	9144.3 (7767.7–10379.7)	−0.84 (−0.93–−0.76) *	−0.33 (−0.97–0.68)	2017–2023
	YLLs	11427.1 (9800.2–12865.1)	8600.9 (7320.7–9776.5)	−0.87 (−0.96–−0.79) *	−0.32 (−0.99–0.75)	2017–2023
	YLDs	592.9 (438.5–764.1)	543.4 (401.6–704.2)	−0.27 (−0.28–−0.26) *	0.29 (0.04–0.53) *	2021–2023
	Incidence	955.5 (881.6–1047.4)	808.7 (748.1–874.4)	−0.51 (−0.52–−0.49) *	0.05 (−0.31–0.29)	2021–2023
	Prevalence	9453.9 (8666.5–10334.3)	8990.1 (8334.8–9732.2)	−0.16 (−0.17–−0.14) *	0.06 (−0.37–0.32)	2021–2023
**JORDAN**						
	Deaths	422.4 (350.7–476.8)	224.7 (200.4–247.6)	−1.84 (−2.02–−1.72) *	−1.97 (−3.56–0.85)	2012–2023
	DALYs	9475.8 (7824.6–10760.0)	4680.5 (4234.2–5094.4)	−2.13 (−2.24–−2.02) *	−5.17 (−7.34–−3.03) *	2021–2023
	YLLs	8970.0 (7327.8–10235.2)	4285.4 (3878.8–4685.5)	−2.23 (−2.35–−2.11) *	−5.54 (−7.81–−2.90) *	2021–2023
	YLDs	505.8 (371.3–649.3)	395.1 (303.7–519.4)	−0.76 (−0.78–−0.74) *	−1.16 (−1.34–−0.60) *	2014–2023
	Incidence	814.7 (760.7–880.0)	536.2 (500.1–592.1)	−1.27 (−1.30–−1.25) *	−0.45 (−0.94–0.32)	2020–2023
	Prevalence	7882.8 (7342.5–8562.9)	6254.9 (5801.3–6813.5)	−0.70 (−0.72–−0.69) *	−0.71 (−1.07–−0.42) *	2021–2023
**KUWAIT**						
	Deaths	413.7 (383.1–435.2)	187.4 (170.1–203.7)	−2.33 (−2.53–−2.14) *	−1.77 (−5.09–1.29)	2014–2023
	DALYs	8495.6 (8008.2–8945.4)	3891.0 (3608.8–4188.8)	−2.35 (−2.56–−2.13) *	−1.88 (−2.90–0.52)	2014–2023
	YLLs	7986.5 (7597.1–8370.4)	3443.7 (3190.9–3677.1)	−2.53 (−2.77–−2.28) *	−2.05 (−3.23–2.51)	2014–2023
	YLDs	509.1 (379.2–643.2)	447.3 (334.9–582.6)	−0.40 (−0.44–−0.37) *	0.14 (−0.59–0.62)	2021–2023
	Incidence	874.2 (799.3–941.6)	623.5 (570.4–681.0)	−1.02 (−1.06–−0.99) *	0.34 (−0.38–0.92)	2021–2023
	Prevalence	8560.7 (7905.5–9442.5)	7378.4 (6874.2–7959.0)	−0.45 (−0.48–−0.43) *	0.51 (−0.25–0.95)	2021–2023
**LEBANON**						
	Deaths	162.0 (113.9–209.7)	129.9 (109.1–152.4)	−0.47 (−1.59–0.69)	0.42 (−15.02–22.29)	2003–2023
	DALYs	3972.6 (2917.3–4993.8)	3136.3 (2656.4–3571.0)	−0.56 (−1.79–0.65)	0.68 (−0.42–23.22)	2004–2023
	YLLs	3488.6 (2450.1–4540.8)	2703.1 (2295.6–3135.4)	−0.60 (−2.00–0.78)	0.82 (−0.44–26.91)	2004–2023
	YLDs	484.0 (359.1–626.9)	433.3 (319.5–568.6)	−0.33 (−0.35–−0.31) *	−0.03 (−0.48–0.30)	2021–2023
	Incidence	661.9 (606.7–739.1)	485.2 (449.3–537.5)	−0.94 (−0.95–−0.93) *	0.17 (0.04–0.36) *	2019–2023
	Prevalence	7772.9 (7220.5–8514.2)	7225.6 (6597.2–7848.3)	−0.22 (−0.24–−0.21) *	−0.03 (−0.33–0.42)	2020–2023
**LIBYA**						
	Deaths	434.1 (396.7–476.3)	368.0 (315.6–411.1)	−0.52 (−0.69–−0.35) *	−3.53 (−6.25–0.71)	2021–2023
	DALYs	10067.5 (9023.7–11194.3)	7667.8 (6523.4–8756.9)	−0.85 (−1.01–−0.69) *	−3.88 (−6.75–0.83)	2021–2023
	YLLs	9559.5 (8547.8–10697.8)	7088.6 (5908.4–8167.3)	−0.93 (−1.10–−0.76) *	−4.18 (−7.24–0.93)	2021–2023
	YLDs	508.0 (377.6–653.4)	579.2 (436.2–768.0)	0.40 (0.38–0.41) *	−0.04 (−0.39–0.22)	2021–2023
	Incidence	673.6 (619.3–729.2)	663.6 (613.9–725.5)	−0.05 (−0.07–−0.03) *	−0.17 (−0.37–0.14)	2019–2023
	Prevalence	8153.6 (7577.7–8838.8)	9254.6 (8630.5–10005.8)	0.38 (0.36–0.39) *	−0.26 (−0.55–0.01)	2015–2023
**MOROCCO**						
	Deaths	786.8 (729.7–841.8)	546.0 (466.3–623.2)	−1.09 (−1.13–−1.05) *	−1.07 (−1.70–−0.28) *	2021–2023

	DALYs	15796.2 (14492.1–17085.8)	10445.5 (8954.9–11683.7)	−1.23 (−1.27–−1.20) *	−1.95 (−2.68–−1.25) *	2021–2023
	YLLs	15296.1 (13994.8–16609.7)	10029.1 (8521.7–11289.5)	−1.26 (−1.30–−1.23) *	−2.02 (−2.77–−1.28) *	2021–2023
	YLDs	500.1 (373.2–647.1)	416.3 (309.6–539.6)	−0.55 (−0.57–−0.54) *	−0.20 (−0.55–0.03)	2021–2023
	Incidence	798.6 (741.7–881.1)	604.8 (560.4–658.9)	−0.84 (−0.86–−0.83) *	−0.43 (−0.93–−0.07) *	2021–2023
	Prevalence	8024.8 (7482.7–8776.0)	6803.7 (6321.4–7385.7)	−0.50 (−0.52–−0.48) *	−0.44 (−0.73–0.08)	2020–2023
**OMAN**						
	Deaths	524.0 (468.7–578.7)	274.1 (230.6–315.6)	−1.87 (−2.11–−1.55) *	−8.52 (−12.43–−3.32) *	2021–2023
	DALYs	11919.6 (10602.4–13239.8)	5472.5 (4689.2–6340.5)	−2.28 (−2.50–−1.99) *	−6.55 (−10.25–−2.81) *	2021–2023
	YLLs	11294.2 (9957.4–12665.2)	4946.3 (4194.7–5782.4)	−2.42 (−2.65–−2.09) *	−7.19 (−11.18–−2.96) *	2021–2023
	YLDs	625.4 (462.3–802.1)	526.2 (389.7–700.6)	−0.53 (−0.54–−0.52) *	−0.23 (−0.54–−0.03) *	2021–2023
	Incidence	792.1 (729.4–859.5)	556.0 (515.1–608.0)	−1.08 (−1.11–−1.05) *	−0.04 (−0.57–0.89)	2020–2023
	Prevalence	10289.0 (9354.6–11399.6)	8563.7 (7820.0–9405.3)	−0.55 (−0.57–−0.55) *	−0.37 (−0.70–−0.19) *	2021–2023
**PALESTINE**						
	Deaths	500.5 (430.0–553.8)	283.4 (254.8–308.8)	−1.76 (−1.89–−1.67) *	−0.44 (−2.85–2.20)	2020–2023
	DALYs	10530.0 (8905.4–11798.0)	5610.1 (5123.5–6065.0)	−1.93 (−2.09–−1.79) *	0.78 (−2.57–3.07)	2021–2023
	YLLs	10000.3 (8384.2–11249.4)	5132.1 (4726.0–5569.0)	−2.04 (−2.23–−1.88) *	0.97 (−2.81–3.39)	2021–2023
	YLDs	529.8 (396.9–678.9)	478.1 (357.2–613.6)	−0.30 (−0.32–−0.27) *	−0.68 (−1.08–−0.42) *	2019–2023
	Incidence	879.4 (811.2–951.1)	647.7 (604.8–708.4)	−0.93 (−0.95–−0.91) *	−0.10 (−0.56–0.56)	2020–2023
	Prevalence	8827.4 (8145.9–9639.8)	7872.5 (7356.2–8435.1)	−0.34 (−0.36–−0.31) *	−1.52 (−1.90–−0.85) *	2021–2023
**QATAR**						
	Deaths	453.1 (373.1–529.1)	160.2 (129.0–190.5)	−3.27 (−3.80–−2.76) *	7.66 (−2.03–16.50)	2021–2023
	DALYs	8868.3 (7411.4–10303.7)	3150.7 (2595.4–3660.7)	−3.21 (−3.67–−2.82) *	8.34 (−1.38–14.39)	2021–2023
	YLLs	8352.1 (6900.5–9760.4)	2714.2 (2237.9–3189.5)	−3.51 (−4.08–−3.01) *	9.71 (−2.11–17.11)	2021–2023
	YLDs	516.2 (385.2–663.1)	436.5 (325.8–581.2)	−0.51 (−0.53–−0.50) *	−0.51 (−0.84–−0.33) *	2019–2023
	Incidence	854.9 (786.3–939.1)	496.9 (455.2–547.4)	−1.63 (−1.69–−1.58) *	−0.11 (−0.81–1.31)	2019–2023
	Prevalence	8504.2 (7854.6–9355.6)	6996.3 (6440.7–7561.7)	−0.59 (−0.60–−0.58) *	−0.29 (−0.33–−0.26) *	2015–2023
**SAUDI ARABIA**						
	Deaths	498.0 (438.2–559.8)	350.4 (288.4–398.2)	−1.03 (−1.20–−0.88) *	−4.12 (−6.67–−0.27) *	2021–2023
	DALYs	9958.9 (8565.6–11239.6)	6613.4 (5570.9–7461.0)	−1.21 (−1.35–−1.02) *	−3.60 (−6.29–−0.31) *	2021–2023
	YLLs	9572.1 (8224.4–10904.6)	6197.8 (5123.2–6998.0)	−1.29 (−1.44–−1.08) *	−3.86 (−6.68–−0.35) *	2021–2023
	YLDs	386.8 (289.2–496.8)	415.6 (310.9–550.0)	0.23 (0.21–0.24) *	0.54 (0.28–1.01) *	2020–2023
	Incidence	672.7 (613.7–755.7)	629.5 (577.1–689.5)	−0.20 (−0.22–−0.18) *	−0.07 (−0.33–0.38)	2019–2023
	Prevalence	6311.1 (5882.0–6897.1)	6968.8 (6441.8–7680.4)	0.31 (0.29–0.34) *	0.73 (0.46–1.33) *	2019–2023
**SUDAN**						
	Deaths	933.6 (854.1–1006.1)	575.0 (493.4–655.3)	−1.43 (−1.51–−1.36) *	−0.66 (−1.47–0.93)	2019–2023
	DALYs	20616.1 (18337.5–22672.1)	11646.7 (9685.8–13600.2)	−1.70 (−1.79–−1.61) *	−0.79 (−1.71–0.96)	2019–2023
	YLLs	20028.1 (17710.2–22033.4)	11083.0 (9112.2–12968.8)	−1.76 (−1.86–−1.67) *	−0.82 (−1.78–1.00)	2019–2023
	YLDs	588.0 (435.6–755.9)	563.8 (419.0–737.1)	−0.13 (−0.14–−0.12) *	0.18 (−0.10–0.34)	2021–2023
	Incidence	966.3 (884.5–1065.7)	794.0 (736.6–854.1)	−0.59 (−0.60–−0.58) *	0.20 (0.06–0.51) *	2020–2023
	Prevalence	9536.3 (8774.6–10499.1)	9106.1 (8468.8–9915.9)	−0.14 (−0.15–−0.13) *	0.13 (−0.13–0.28)	2021–2023
**SYRIA**						
	Deaths	697.1 (627.3–753.0)	489.4 (429.6–547.3)	−1.07(−1.15–−0.99) *	0.97(−0.80–2.45)	2021–2023
	DALYs	14850.8 (13298.5–16131.7)	8435.6 (7462.9–9306.9)	−1.69(−1.78–1.62) *	0.35 (−1.27–1.69)	2021–2023
	YLLs	14235.6 (12721.4–15502.4)	7900.2 (7004.8–8753.4)	−1.76 (−1.86–−1.68) *	1.05 (−0.81–2.59)	2021–2023
	YLDs	615.2 (456.7–790.3)	535.3 (400.0–697.4)	−0.42 (−0.43–−0.41) *	−0.15 (−0.41–0.03)	2021–2023
	Incidence	1088.1 (1010.3–1173.3)	785.6 (724.6–855.0)	−0.99 (−1.00–−0.97) *	−0.26(−0.66–−0.05) *	2021–2023
	Prevalence	10107.2 (9390.2–11029.8)	9200.5 (8536.2–10072.3)	−0.28 (−0.29–−0.27) *	−0.17 (−0.32–0.06)	2020–2023
**TUNISIA**						
	Deaths	319.1 (247.8–381.4)	172.0 (142.9–198.9)	−1.92 (−2.10–−1.68) *	5.01 (0.97–9.12) *	2021–2023
	DALYs	6078.7 (4838.6–7376.1)	3650.7 (3148.1–4094.9)	−1.57 (−1.74–−1.41) *	6.34 (2.50–9.40) *	2021–2023
	YLLs	5656.4 (4451.4–6927.8)	3247.4 (2779.7–3705.5)	−1.70 (−1.88–−1.55) *	7.20 (3.12–10.71) *	2021–2023
	YLDs	422.3 (313.6–542.8)	403.3 (295.1–534.4)	−0.15 (−0.16–−0.13) *	−0.29 (−0.54–−0.04) *	2014–2023
	Incidence	597.5 (551.9–662.1)	459.9 (424.1–509.8)	−0.78 (−0.81–−0.76) *	0.51 (0.05–1.21) *	2020–2023
	Prevalence	6651.2 (6144.1–7279.2)	6529.9 (6010.8–7146.3)	−0.06 (−0.08–−0.05) *	−0.14 (−0.39–0.07)	2015–2023
**TÜRKIYE**						
	Deaths	442.0 (364.2–504.2)	219.3 (183.3–253.5)	−2.08 (−2.25–−1.84) *	−5.07 (−7.69–−1.84) *	2021–2023
	DALYs	9261.8 (7512.8–10593.7)	4197.9 (3577.0–4796.2)	−2.36 (−2.52–−2.15) *	−4.46 (−7.32–−1.66) *	2021–2023
	YLLs	8762.4 (7103.5–10098.6)	3765.8 (3179.7–4353.4)	−2.51 (−2.69–−2.29) *	−4.95 (−8.04–−1.83) *	2021–2023
	YLDs	499.4 (372.5–658.6)	432.1 (324.9–565.9)	−0.45 (−0.46–−0.43) *	0.00 (−0.18–0.30)	2015–2023
	Incidence	710.2 (656.5–773.1)	526.8 (486.8–575.7)	−0.91 (−0.93–−0.89) *	0.32 (0.01–0.82) *	2020–2023
	Prevalence	8062.0 (7481.3–8851.1)	7242.2 (6640.9–7787.0)	−0.34 (−0.37–−0.30) *	−0.24 (−0.89–0.18)	2019–2023
**UNITED ARAB EMIRATES**						
	Deaths	356.8 (213.3–484.2)	217.3 (169.5–258.6)	−1.55 (−1.71–−1.40) *	−3.41 (−5.43–−0.34) *	2017–2023
	DALYs	7570.7 (4334.2–10320.3)	3669.9 (2971.2–4224.6)	−2.18 (−2.41–−2.04) *	0.00 (−4.37–3.30)	2021–2023
	YLLs	7029.9 (3854.2–9715.6)	3254.3 (2597.8–3782.1)	−2.32 (−2.58–−2.16) *	0.11 (−4.83–3.81)	2021–2023
	YLDs	540.8 (402.9–699.2)	415.6 (309.5–538.3)	−0.80 (−0.82–−0.78) *	−0.78 (−1.12–−0.61) *	2020–2023
	Incidence	827.7 (759.8–914.3)	466.6 (430.0–520.9)	−1.73 (−1.76–−1.69) *	−0.02 (−0.38–0.29)	2019–2023
	Prevalence	8535.2 (7947.8–9321.0)	6474.7 (5938.7–6993.2)	−0.83 (−0.85–−0.82) *	−1.13 (−1.37–−0.86) *	2021–2023
**YEMEN**						
	Deaths	793.2 (730.0–862.5)	483.8 (436.0–541.7)	−1.45 (−1.55–−1.37) *	−0.35 (−1.17–2.23)	2018–2023
	DALYs	16555.3 (14822.1–18259.8)	9446.0 (8391.5–10567.6)	−1.67 (−1.78–−1.59) *	−0.66 (−1.58–1.93)	2018–2023
	YLLs	16007.7 (14340.1–17698.4)	8928.5 (7929.8–10027.0)	−1.73 (−1.85–−1.65) *	−0.68 (−1.64–2.07)	2018–2023
	YLDs	547.6 (409.1–705.9)	517.5 (382.7–678.2)	−0.17 (−0.18–−0.16) *	0.14 (−0.03–0.28)	2021–2023
	Incidence	916.9 (849.8–1012.7)	743.9 (685.1–809.2)	−0.63 (−0.64–−0.62) *	−0.21 (−0.33–−0.04) *	2019–2023
	Prevalence	9066.4 (8330.5–10102.7)	8617.8 (7947.9–9385.8)	−0.15 (−0.16–−0.15) *	0.00 (−0.25–0.16)	2021–2023

Abbreviations: AAPC, average annual percentage change; APC, annual percentage change; ASR, age-standardised rate; CI, confidence interval; DALY, disability-adjusted life year; NAME, North Africa and Middle East; UI, uncertainty interval; YLD, years lived with disability; YLL, years of life lost.

* APC and AAPC were considered statistically significant if $p < 0.05$ and the 95% CI did not include zero.

Table S3. Joinpoint regression results for cardiovascular disease incidence, mortality, and DALYs in the NAME region by sex, 1990–2023.

Outcome	Sex	Period (years)	APC (95% CI)
**INCIDENCE**			
	Both	1990–1997	−0.05 (−0.12 to 0.06)
		1997–2001	−0.36 (−1.02 to −0.13) *
		2001–2010	−1.05 (−1.72 to −0.97) *
		2010–2015	−1.71 (−2.06 to −0.67) *
		2015–2021	−0.61 (−0.74 to −0.53) *
		**2021–2023**	**0.06 (−0.38 to 0.34)**
	Male	1990–1996	0.30 (0.03 to 0.54) *
		1996–2001	−0.05 (−0.28 to 0.49)
		2001–2006	−0.83 (−0.96 to 0.07)
		2006–2010	−1.04 (−1.84 to −0.79) *
		2010–2015	−1.59 (−1.90 to −0.47) *
		2015–2021	−0.62 (−1.68 to −0.49) *
		**2021–2023**	**−0.19 (−0.56 to 0.06)**
	Female	1990–1997	−0.37 (−0.44 to −0.08) *
		1997–2001	−0.68 (−1.07 to −0.45) *
		2001–2010	−1.18 (−1.31 to −1.11) *
		2010–2015	−1.79 (−2.11 to −1.61) *
		2015–2020	−0.70 (−0.95 to −0.52) *
		**2020–2023**	**0.12 (−0.14 to 0.50)**
**PREVALENCE**			
	Both	1990–1995	−0.14 (−0.40 to −0.03) *
		1995–2000	0.18 (−0.05 to 0.41)
		2000–2005	0.46 (−0.09 to 0.69)
		2005–2009	−0.07 (−0.72 to 0.19)
		2009–2020	−0.59 (−0.68 to −0.53) *
		**2020–2023**	**−0.24 (−0.47 to 0.07)**
	Male	1990–1994	−0.02 (−0.26 to 0.10)
		1994–2000	0.15 (0.06 to 0.52) *
		2000–2006	0.41 (−0.08 to 0.60)
		2006–2010	−0.01 (−0.98 to 0.12)
		2010–2015	−0.88 (−1.10 to −0.21) *
		**2015–2023**	**−0.35 (−0.55 to −0.17) ***
	Female	1990–1995	−0.32 (−0.61 to −0.20) *
		1995–2000	0.22 (−0.14 to 0.43)
		2000–2005	0.53 (−0.46 to 0.71)
		2005–2016	−0.41 (−0.45 to −0.36) *
		2016–2020	−0.90 (−1.13 to −0.69) *
		**2020–2023**	**−0.22 (−0.48 to 0.19)**
**DEATHS**			
	Both	1990–2007	−1.41 (−2.29 to −1.15) *
		2007–2011	−2.66 (−3.64 to −0.87) *
		2011–2015	−0.67 (−2.98 to 0.38)
		2015–2019	−2.00 (−3.01 to −0.93) *
		**2019–2023**	**−0.33 (−1.37 to 1.75)**
	Male	1990–2006	−1.19 (−2.11 to −0.07) *
		2006–2011	−2.23 (−3.18 to −0.63) *
		2011–2015	−0.77 (−2.94 to 0.06)
		2015–2019	−1.82 (−2.67 to −1.17) *
		**2019–2023**	**−0.28 (−0.88 to 1.14)**
	Female	1990–2007	−1.57 (−2.60 to 0.05)
		2007–2011	−2.86 (−4.02 to −0.81) *
		2011–2015	−0.70 (−3.63 to 0.47)
		2015–2019	−2.16 (−3.36 to −0.94) *
		**2019–2023**	**−0.34 (−1.51 to 1.97)**
**DALYs**			
	Both	1990–2006	−1.58 (−2.59 to −0.36) *
		2006–2011	−2.41 (−3.28 to −0.96) *
		2011–2015	−1.09 (−3.13 to −0.23) *
		2015–2020	−2.01 (−3.00 to −1.25) *
		**2020–2023**	**0.21 (−0.90 to 2.18)**
	Male	1990–1999	−1.32 (−2.89 to 1.01)
		1999–2020	−1.67 (−2.93 to −0.30) *
		**2020–2023**	**0.01 (−1.58 to 2.23)**
	Female	1990–2006	−1.71 (−2.96 to −0.26) *
		2006–2011	−2.69 (−3.80 to −0.63) *
		2011–2015	−1.18 (−3.53 to −0.17) *
		2015–2020	−2.10 (−3.26 to −1.21) *
		**2020–2023**	**0.28 (−1.01 to 2.53)**

* APC was considered statistically significant if $p < 0.05$ and the 95% CI did not include zero. APC, annual percentage change; CI, confidence interval; DALY, disability-adjusted life year; NAME, North Africa and Middle East.

Table S4. Regional trends in age-standardised rates of cardiovascular burden stratified by specific cause, both sexes, 1990–2023.

Cause	Measure	ASR 1990 (95% UI)	ASR 2023 (95% UI)	AAPC (95% CI)	Recent APC (95% CI)	Recent Period
**AA**						
	Deaths	0.54 (0.34–0.85)	0.93 (0.76–1.10)	1.71 (1.60–1.82) *	−0.68 (−3.05–0.83)	2020–2023
	DALYs	13.57 (8.47–22.21)	20.96 (17.04–25.00)	1.33 (1.21–1.46) *	−0.71 (−3.16–0.82)	2020–2023
	YLLs	13.57 (8.47–22.21)	20.96 (17.04–25.00)	1.33 (1.21–1.46) *	−0.71 (−3.16–0.82)	2020–2023
**AF/AFL**						
	Deaths	2.87 (2.22–3.60)	4.42 (3.40–5.46)	1.35 (1.28–1.41) *	1.56 (1.32–1.87) *	2010–2023
	DALYs	68.58 (54.92–84.94)	87.25 (68.57–108.74)	0.75 (0.72–0.80) *	1.19 (0.73–2.03) *	2018–2023
	YLLs	37.96 (29.80–47.00)	54.52 (42.92–66.89)	1.12 (1.06–1.20) *	1.39 (−0.64–2.34)	2010–2023
	YLDs	30.62 (19.46–43.88)	32.73 (20.57–47.77)	0.21 (0.18–0.24) *	1.27 (0.84–2.18) *	2020–2023
	Incidence	39.03 (30.00–49.73)	41.60 (31.84–53.77)	0.20 (0.18–0.21) *	0.76 (0.52–1.12) *	2020–2023
	Prevalence	408.91 (308.81–521.42)	439.10 (328.39–560.42)	0.23 (0.19–0.26) *	1.25 (0.81–1.98) *	2020–2023
**CMP**						
	Deaths	3.66 (1.92–6.11)	2.63 (1.79–4.08)	−0.97 (−1.04–−0.89) *	−0.02 (−1.27–1.24)	2018–2023
	DALYs	143.03 (76.59–227.32)	91.60 (59.91–139.39)	−1.33 (−1.39–−1.25) *	0.61 (−0.33–2.51)	2019–2023
	YLLs	140.11 (72.92–224.96)	88.02 (57.29–135.88)	−1.38 (−1.44–−1.30) *	0.61 (−0.31–2.09)	2019–2023
	YLDs	2.92 (2.00–4.21)	3.58 (2.47–5.21)	0.61 (0.58–0.63) *	1.07 (0.44–1.37) *	2021–2023
	Incidence	10.23 (8.05–13.10)	10.23 (8.05–13.13)	0.00 (0.00–0.00)	0.03 (−0.02–0.06)	2019–2023
	Prevalence	33.88 (29.03–39.40)	41.14 (33.67–49.26)	0.59 (0.57–0.60) *	0.78 (0.45–1.20) *	2020–2023
**ENDC**						
	Deaths	0.84 (0.55–1.19)	0.60 (0.48–0.78)	−0.96 (−1.03–−0.88) *	0.23 (−0.32–2.05)	2018–2023
	DALYs	31.93 (20.23–48.75)	19.35 (14.50–26.35)	−1.48 (−1.56–−1.41) *	0.72 (−0.23–2.73)	2019–2023
	YLLs	31.76 (20.11–48.48)	19.13 (14.29–26.11)	−1.50 (−1.58–−1.42) *	0.71 (−0.24–2.75)	2019–2023
	YLDs	0.17 (0.12–0.24)	0.22 (0.15–0.31)	0.76 (0.74–0.80) *	0.81 (0.65–0.99) *	2016–2023
	Incidence	10.81 (9.32–12.41)	13.03 (11.42–14.78)	0.58 (0.56–0.60) *	1.72 (1.29–2.55) *	2020–2023
	Prevalence	2.15 (1.89–2.41)	2.76 (2.44–3.13)	0.76 (0.74–0.79) *	0.84 (0.60–0.99) *	2016–2023
**HHD**						
	Deaths	48.89 (32.00–67.19)	40.75 (31.80–52.45)	−0.53 (−0.64–−0.44) *	0.62 (−0.60–3.12)	2019–2023
	DALYs	898.26 (594.68–1230.98)	699.86 (549.41–898.25)	−0.74 (−0.87–−0.66) *	0.39 (−0.85–2.70)	2019–2023
	YLLs	879.03 (577.52–1219.30)	680.63 (529.94–878.94)	−0.76 (−0.89–−0.68) *	0.42 (−0.86–2.79)	2019–2023
	YLDs	19.23 (12.60–25.39)	19.22 (12.73–26.14)	0.00 (−0.01–0.02)	−0.77 (−1.10–−0.27) *	2021–2023
	Prevalence	239.01 (190.59–298.55)	238.24 (191.00–301.44)	0.00 (−0.02–0.02)	−0.83 (−1.14–−0.33) *	2021–2023
**IHD**						
	Deaths	287.77 (235.93–342.24)	199.43 (175.39–224.36)	−1.05 (−1.14–−0.98) *	0.53 (−0.86–2.61)	2020–2023
	DALYs	6025.14 (4989.47–7129.56)	3992.12 (3530.92–4465.71)	−1.19 (−1.28–−1.11) *	0.77 (−0.61–3.05)	2020–2023
	YLLs	5831.84 (4799.90–6938.18)	3825.75 (3404.37–4302.89)	−1.22 (−1.32–−1.14) *	0.84 (−0.57–3.23)	2020–2023
	YLDs	193.30 (138.86–263.26)	166.37 (122.02–224.82)	−0.45 (−0.47–−0.44) *	−0.60 (−0.92–−0.36) *	2021–2023
	Incidence	429.91 (385.77–482.97)	296.31 (258.45–335.12)	−1.14 (−1.18–−1.11) *	−0.51 (−1.34–−0.01) *	2021–2023
	Prevalence	4696.18 (4094.03–5385.86)	4015.35 (3569.07–4497.30)	−0.47 (−0.50–−0.45) *	−0.76 (−1.19–−0.43) *	2021–2023
**PAD-LE**						
	Deaths	0.27 (0.18–0.38)	0.36 (0.29–0.43)	0.87 (0.75–1.04) *	−2.28 (−4.43–0.67)	2021–2023
	DALYs	10.92 (7.01–16.70)	11.67 (8.17–16.79)	0.19 (0.14–0.24) *	−0.70 (−1.92–−0.42) *	2014–2023
	YLLs	5.12 (3.35–7.44)	5.73 (4.72–6.96)	0.41 (0.23–0.56) *	−0.28 (−2.37–2.28)	2018–2023
	YLDs	5.80 (2.66–10.97)	5.94 (2.68–11.42)	0.07 (0.05–0.09) *	−0.61 (−1.01–−0.47) *	2018–2023
	Incidence	101.10 (77.13–129.44)	115.50 (88.16–148.05)	0.41 (0.40–0.42) *	−0.22 (−0.44–−0.07) *	2020–2023
	Prevalence	920.42 (692.22–1182.54)	1068.62 (810.04–1372.70)	0.46 (0.44–0.47) *	−0.14 (−0.46–−0.03) *	2019–2023
**NRVHD**						
	Deaths	1.71 (1.13–2.39)	1.77 (1.26–2.43)	0.13 (0.04–0.21) *	0.24 (−1.24–1.59)	2018–2023
	DALYs	44.82 (29.63–62.86)	43.17 (31.33–58.28)	−0.10 (−0.14–−0.04) *	0.23 (−0.15–1.38)	2018–2023
	YLLs	43.27 (28.19–61.78)	41.50 (29.48–56.61)	−0.11 (−0.16–−0.05) *	0.25 (−0.24–1.46)	2018–2023
	YLDs	1.55 (0.95–2.56)	1.66 (0.99–2.89)	0.23 (0.21–0.25) *	0.09 (−0.36–0.70)	2020–2023
	Incidence	8.85 (7.93–9.95)	9.75 (8.71–10.82)	0.30 (0.29–0.32) *	0.16 (0.00–0.36)	2019–2023
	Prevalence	106.31 (93.34–121.38)	119.86 (104.85–136.45)	0.37 (0.35–0.39) *	0.12 (−0.12–0.57)	2019–2023
**OTHER CVD**						
	Deaths	7.87 (5.27–11.65)	7.55 (5.70–9.73)	−0.09 (−0.15–−0.02) *	1.57 (0.80–2.93) *	2019–2023
	DALYs	345.23 (243.34–489.24)	296.41 (229.03–365.79)	−0.44 (−0.51–−0.38) *	3.25 (1.73–4.30) *	2021–2023
	YLLs	279.99 (184.13–424.08)	215.73 (161.47–278.32)	−0.77 (−0.87–−0.65) *	3.25 (1.69–5.84) *	2020–2023
	YLDs	65.23 (42.65–92.60)	80.68 (51.89–114.37)	0.65 (0.61–0.67) *	0.82 (0.04–1.41) *	2021–2023
	Prevalence	1287.87 (1061.79–1566.10)	1589.52 (1268.31–1969.04)	0.64 (0.60–0.67) *	0.78 (0.04–1.38) *	2021–2023
**PAH**						
	Deaths	0.88 (0.51–1.44)	0.40 (0.25–0.64)	−2.35 (−2.45–−2.28) *	−0.57 (−2.53–0.77)	2021–2023
	DALYs	40.14 (21.84–71.03)	15.56 (9.39–24.94)	−2.83 (−2.92–−2.73) *	0.77 (−0.57–3.05)	2020–2023
	YLLs	39.95 (21.64–70.88)	15.37 (9.21–24.78)	−2.85 (−2.94–−2.75) *	0.78 (−0.58–3.10)	2020–2023
	YLDs	0.19 (0.12–0.27)	0.19 (0.12–0.27)	−0.01 (−0.08–0.06)	−0.19 (−0.94–1.27)	2015–2023
	Incidence	0.56 (0.45–0.67)	0.52 (0.41–0.63)	−0.24 (−0.29–−0.18) *	−0.08 (−0.25–0.07)	2010–2023
	Prevalence	2.02 (1.64–2.52)	2.02 (1.64–2.52)	−0.01 (−0.08–0.06)	−0.19 (−0.94–1.27)	2015–2023
**RHD**						
	Deaths	5.87 (3.42–8.29)	2.19 (1.48–3.06)	−2.93 (−3.05–−2.86) *	−1.85 (−3.26–−1.57) *	2010–2023
	DALYs	260.61 (163.97–353.19)	97.03 (70.45–132.59)	−2.94 (−3.00–−2.86) *	−0.50 (−1.46–0.91)	2020–2023
	YLLs	235.92 (145.08–331.00)	72.59 (47.19–107.21)	−3.48 (−3.59–−3.38) *	−0.03 (−2.30–1.48)	2021–2023
	YLDs	24.69 (15.59–38.07)	24.43 (15.37–38.12)	−0.03 (−0.04–−0.02) *	0.39 (0.20–0.65) *	2020–2023
	Incidence	31.51 (25.28–38.08)	33.21 (26.60–40.29)	0.16 (0.15–0.18) *	0.47 (0.35–0.73) *	2018–2023
	Prevalence	508.42 (412.12–615.41)	510.10 (413.49–614.22)	0.01 (0.00–0.03)	0.35 (0.13–0.61) *	2018–2023
**STROKE**						
	Deaths	218.43 (175.04–264.11)	97.15 (79.71–117.08)	−2.39 (−2.48–−2.31) *	−1.96 (−3.19–−0.38) *	2018–2023
	DALYs	4510.31 (3599.48–5412.35)	1967.60 (1629.08–2369.55)	−2.45 (−2.50–−2.40) *	−1.91 (−2.31–−0.52) *	2018–2023
	YLLs	4321.77 (3401.73–5254.18)	1809.42 (1462.72–2196.19)	−2.58 (−2.65–−2.49) *	−2.04 (−3.80–−0.40) *	2018–2023
	YLDs	188.54 (137.18–236.33)	158.18 (114.87–198.87)	−0.54 (−0.56–−0.53) *	−0.39 (−0.59–−0.10) *	2014–2023
	Incidence	195.71 (179.11–214.91)	135.29 (123.68–148.07)	−1.12 (−1.15–−1.09) *	0.65 (0.30–1.47) *	2019–2023
	Prevalence	1347.23 (1271.85–1438.39)	1135.34 (1085.03–1191.76)	−0.52 (−0.53–−0.51) *	−0.30 (−0.34–−0.25) *	2014–2023

Abbreviations: AA, aortic aneurysm; AAPC, average annual percentage change; AF/AFL, atrial fibrillation and flutter; APC, annual percentage change; ASR, age-standardised rate; CI, confidence interval; CMP, cardiomyopathy and myocarditis; CVD, cardiovascular diseases; DALY, disability-adjusted life year; ENDC, endocarditis; HHD, hypertensive heart disease; IHD, ischaemic heart disease; NRVHD, non-rheumatic valvular heart disease; PAD-LE, lower extremity peripheral arterial disease; PAH, pulmonary arterial hypertension; RHD, rheumatic heart disease; UI, uncertainty interval; YLD, years lived with disability; YLL, years of life lost. * APC and AAPC were considered statistically significant if $p < 0.05$ and the 95% CI did not include zero. Incidence and prevalence estimates for aortic aneurysm were not available in GBD 2023. Incidence estimates for hypertensive heart disease and other cardiovascular disease were not available in GBD 2023.

Figure S1. Time trends in age-standardised rates and all-age numbers of mortality, DALYs, incidence, and prevalence due to cardiovascular diseases in the NAME region, females, 1990–2023.

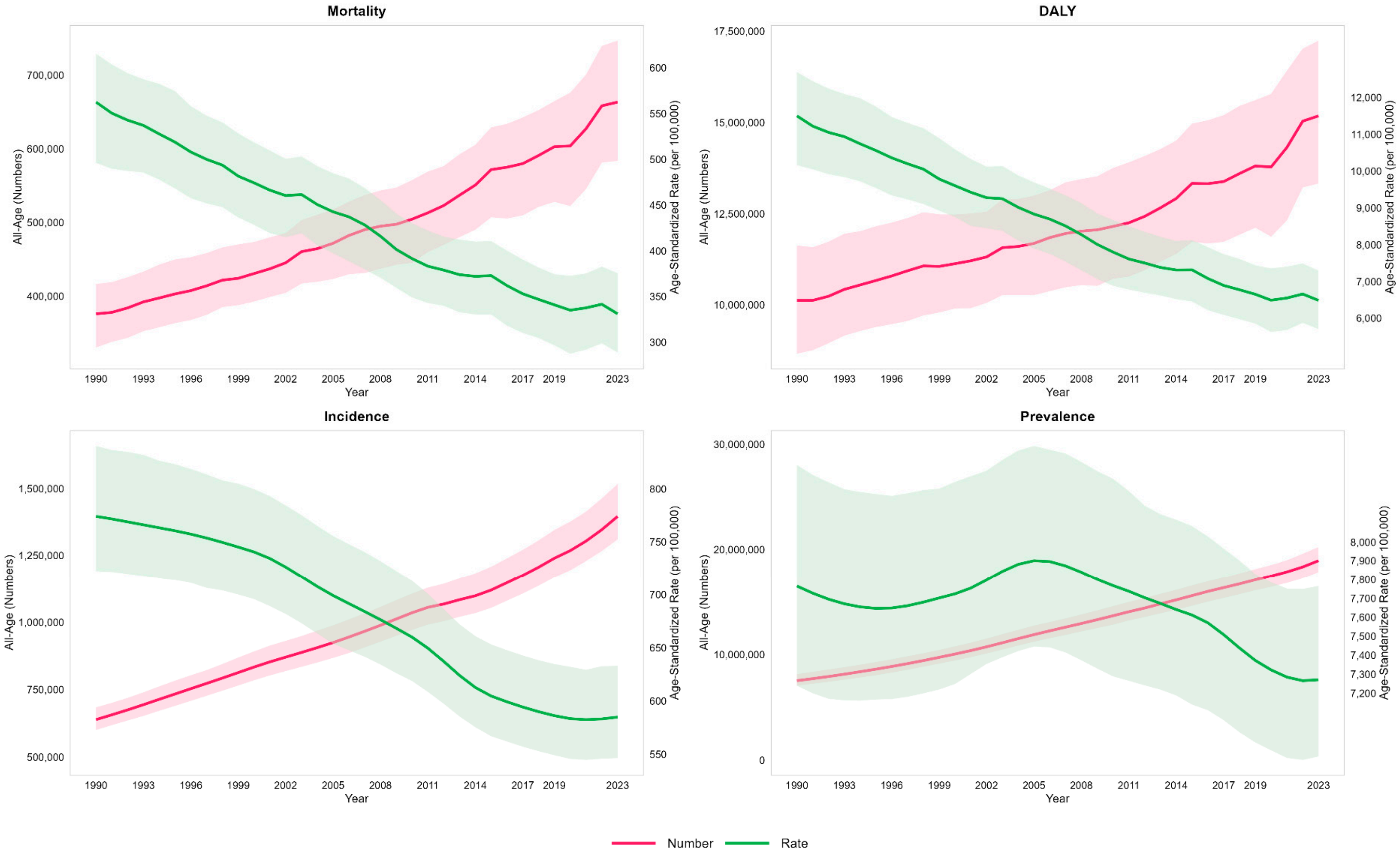

Figure S2. Time trends in age-standardised rates and all-age numbers of mortality, DALYs, incidence, and prevalence due to cardiovascular diseases in the NAME region, males, 1990–2023.

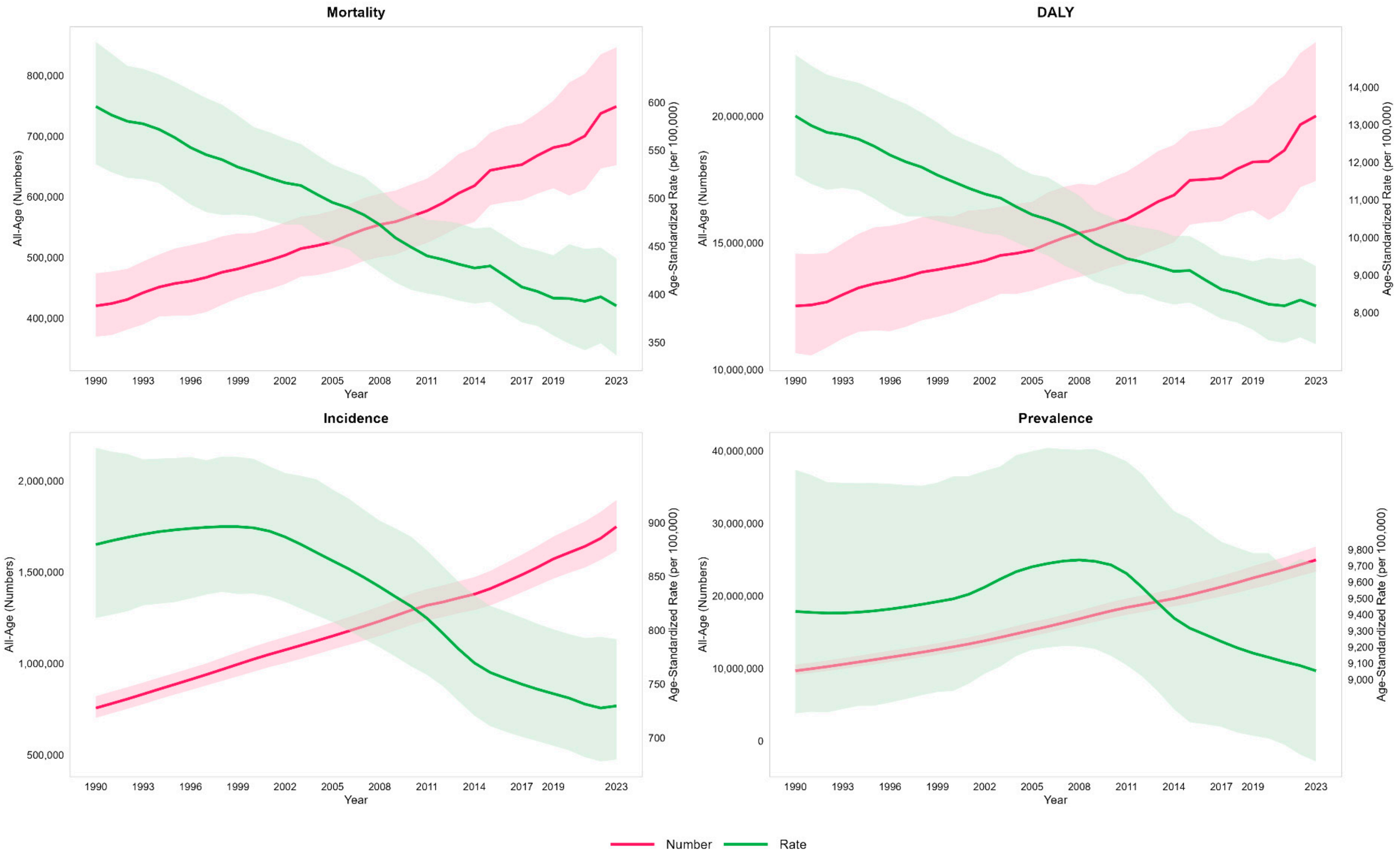

Figure S3. Joinpoint regression segments for cardiovascular disease age-standardised rates in the NAME region, both sexes, 1990 to 2023. Panels: (a) mortality, (b) DALYs, (c) incidence, (d) prevalence. Each panel shows observed annual rates (red squares) and the best-fitting piecewise log-linear model identified by the Joinpoint Regression Program (version 5.4.0.0, NCI). Distinct colours mark successive segments delimited by detected joinpoints; segment-specific annual percent change (APC) values are shown in the legend. APC values flagged with an asterisk (*) are statistically significantly different from zero at alpha = 0.05. The model selection criterion is shown above each panel.

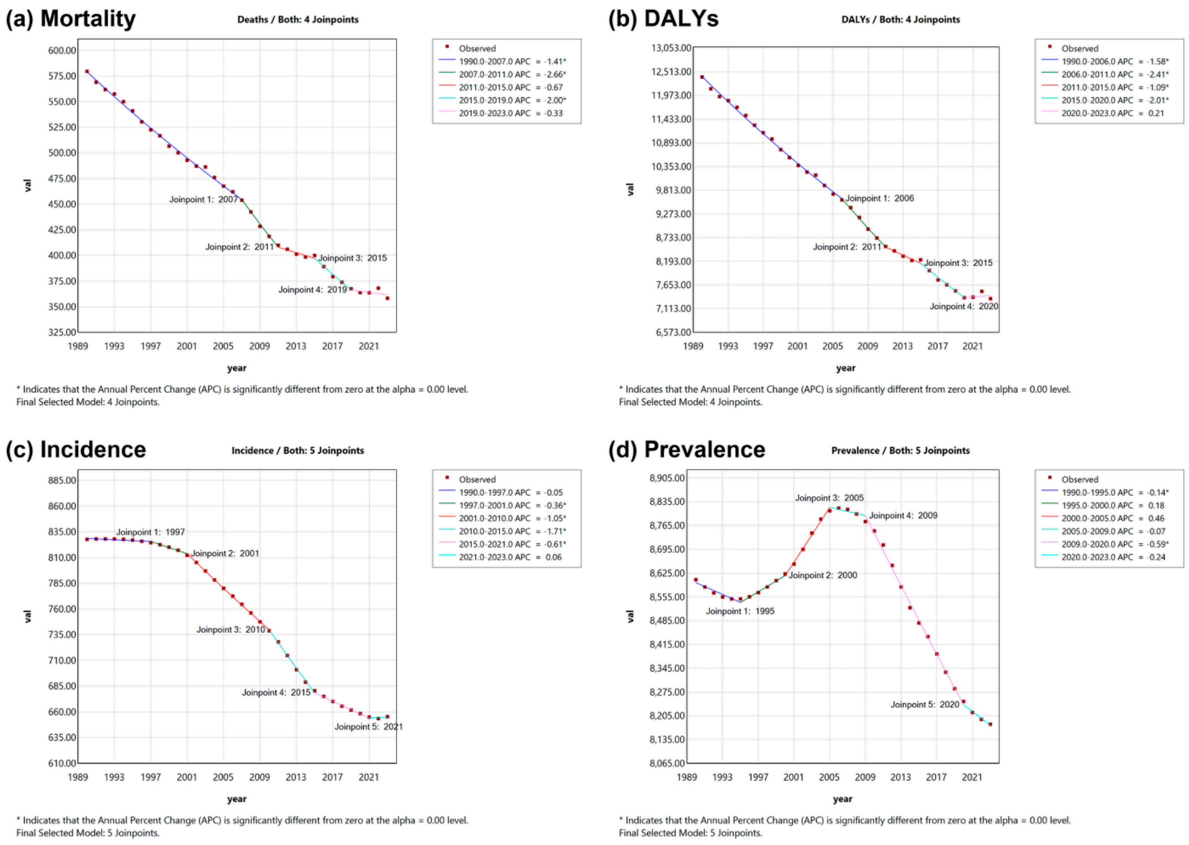

Figure S4. Joinpoint regression segments for cardiovascular disease age-standardised mortality and disability-adjusted life year (DALY) rates by sex in the NAME region, 1990 to 2023. Panels: (a) mortality, males, (b) mortality, females, (c) DALYs, males, (d) DALYs, females. Plot conventions are the same as in Figure S3.

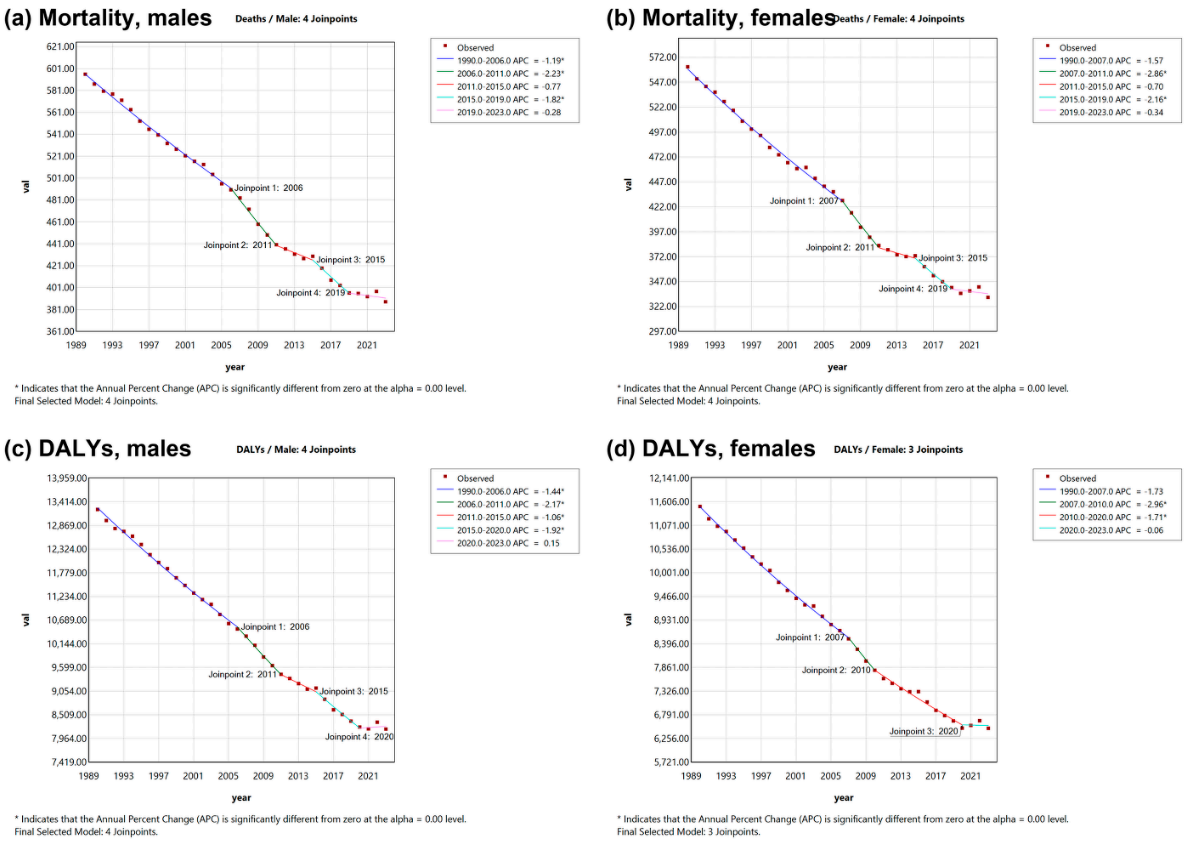

Figure S5. Comparison of CVD risk factors in different NAME countries in terms of DALYs, both sexes, 2023. Abbreviations: CVD, cardiovascular disease; NAME, North Africa and Middle East

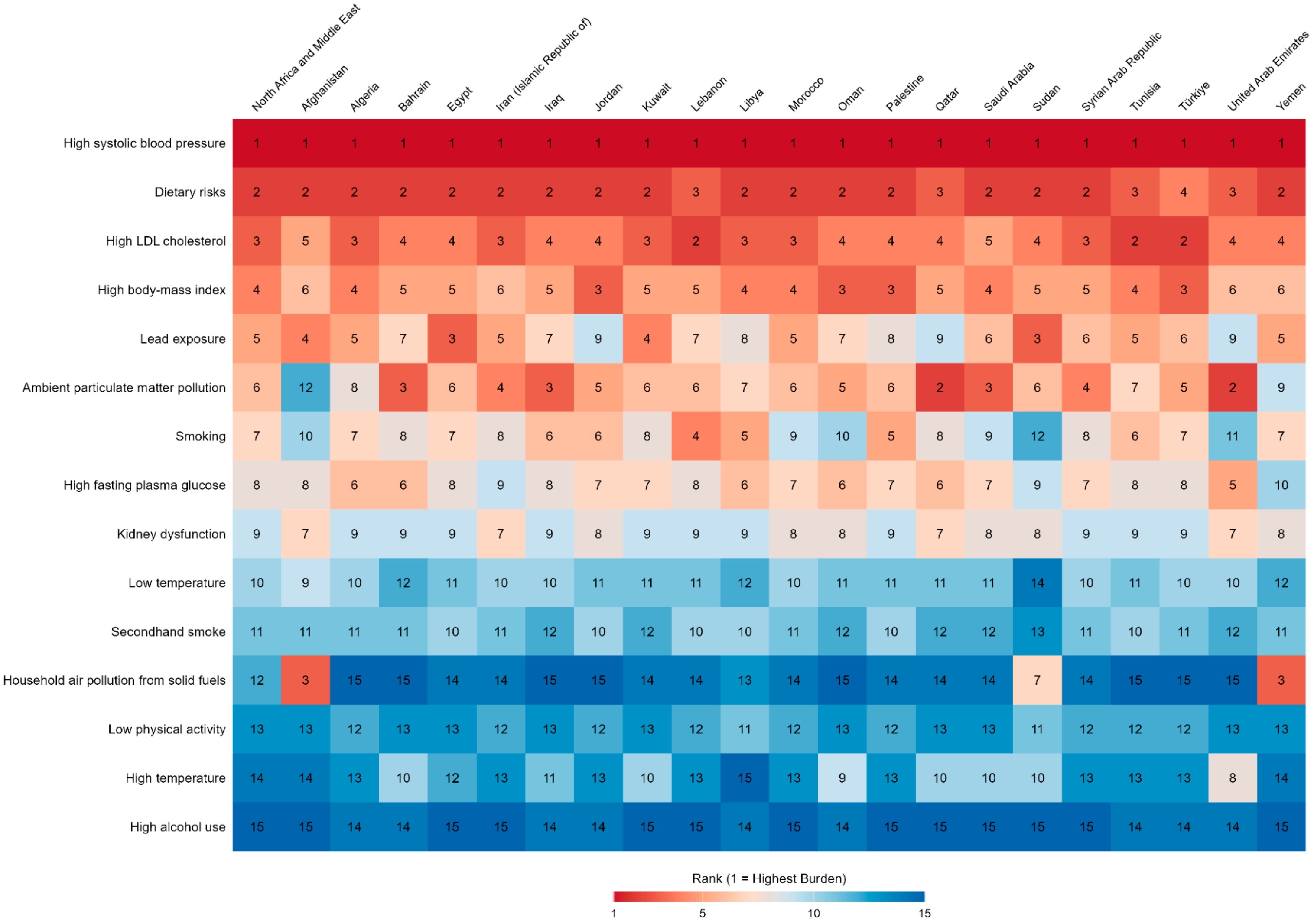

Figure S6. Geographical distribution of age-standardised CVD mortality and DALY rates in the NAME region, 1990 versus 2023. Abbreviations: CVD, cardiovascular disease; DALYs, disability-adjusted life years; NAME, North Africa and Middle East.

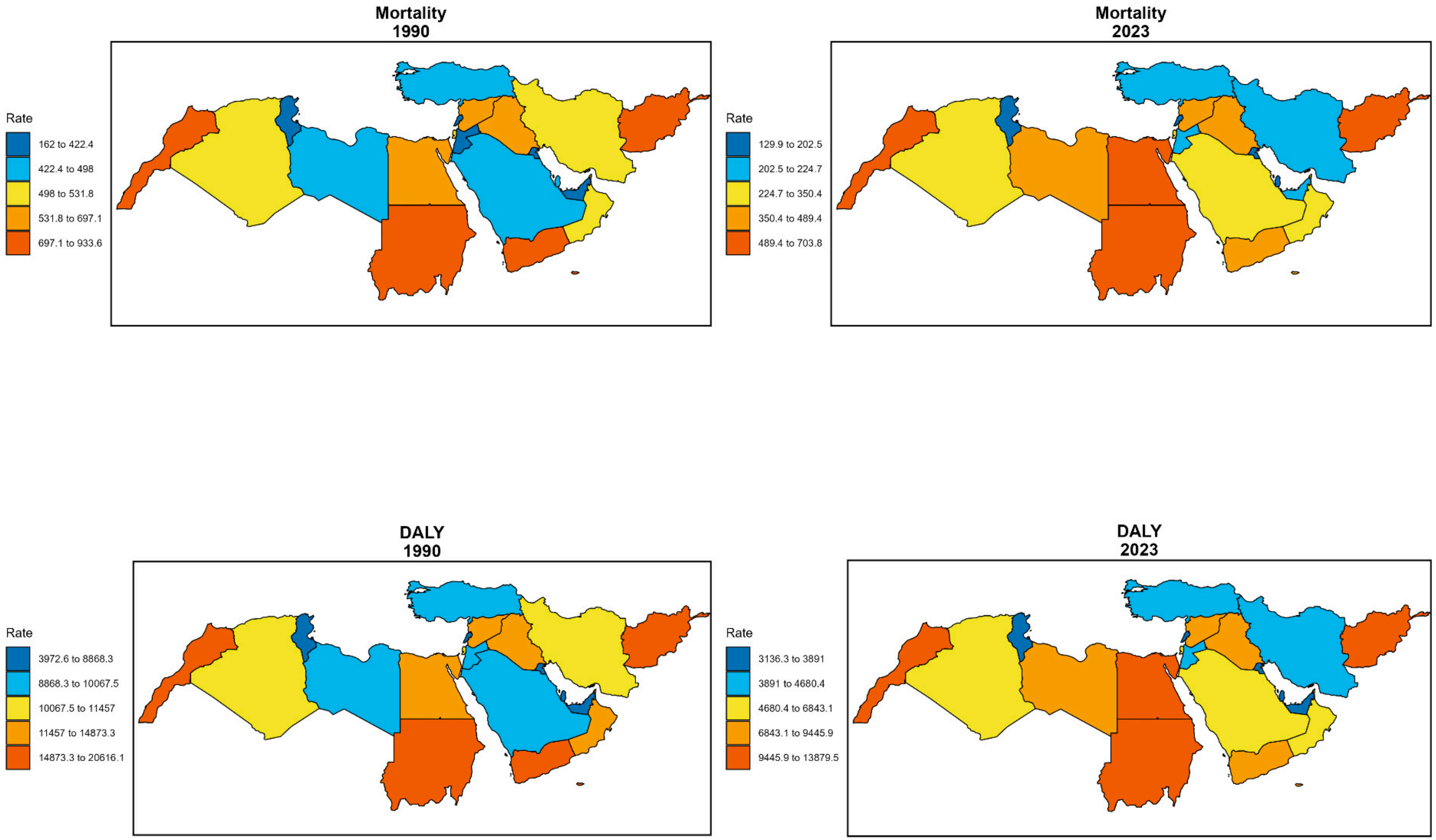

Figure S7. Age-standardised mortality and DALY rates for CVD by age group and sex in the NAME region, 1990 versus 2023. Abbreviations: CVD, cardiovascular disease; DALYs, disability-adjusted life years; NAME, North Africa and Middle East.

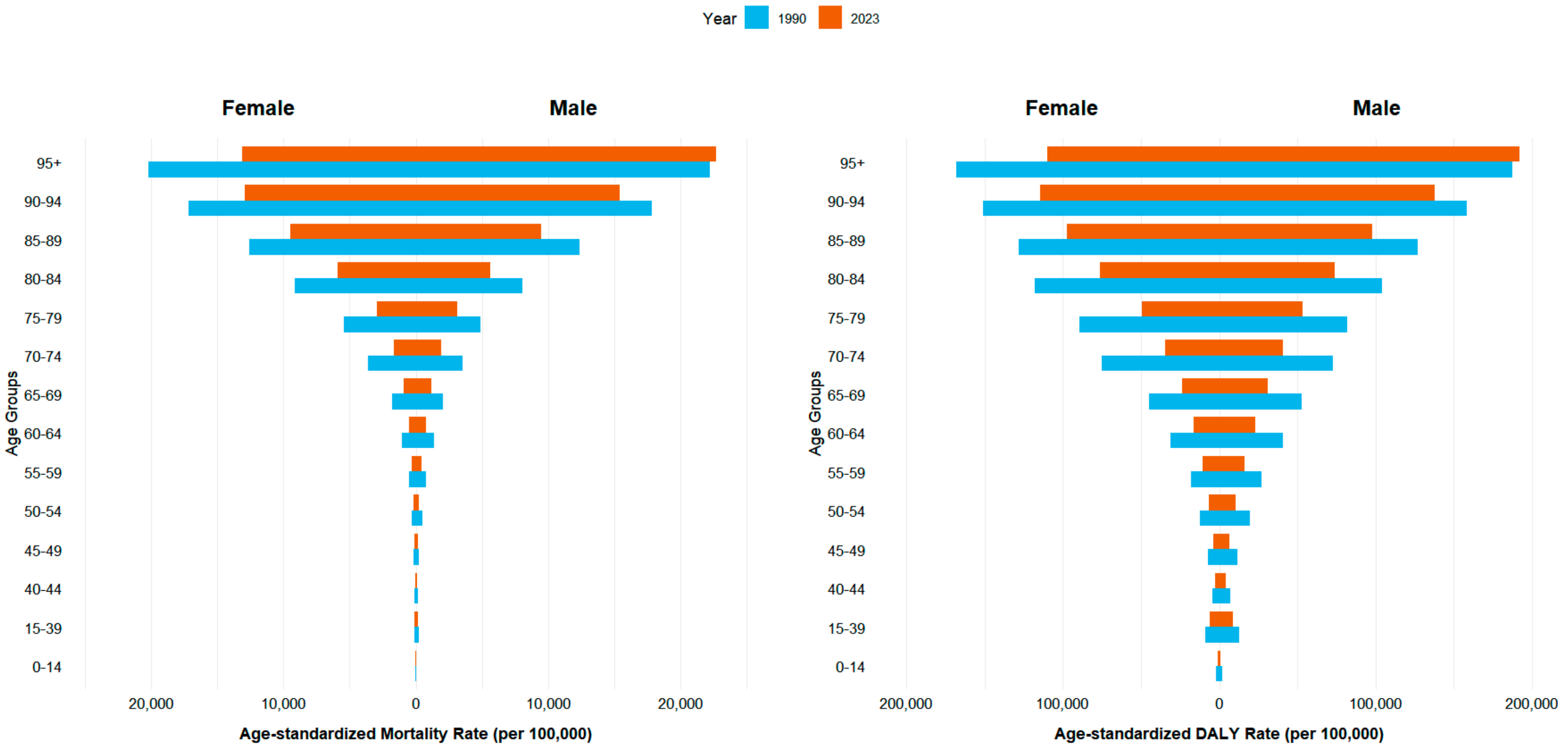

Figure S8. Heatmap of AAPC in DALYs for CVD causes across NAME countries, 1990 to 2023. Abbreviations: AAPC, average annual percentage change; CVD, cardiovascular disease; DALYs, disability-adjusted life years; NAME, North Africa and Middle East; IHD, ischaemic heart disease; STRK, stroke; AF/AFL, atrial fibrillation and flutter; CMP, cardiomyopathy and myocarditis; HHD, hypertensive heart disease; RHD, rheumatic heart disease; NRVHD, non-rheumatic valvular heart disease; AA, aortic aneurysm; LEPAD, lower extremity peripheral arterial disease; ENDC, endocarditis; PAH, pulmonary arterial hypertension.

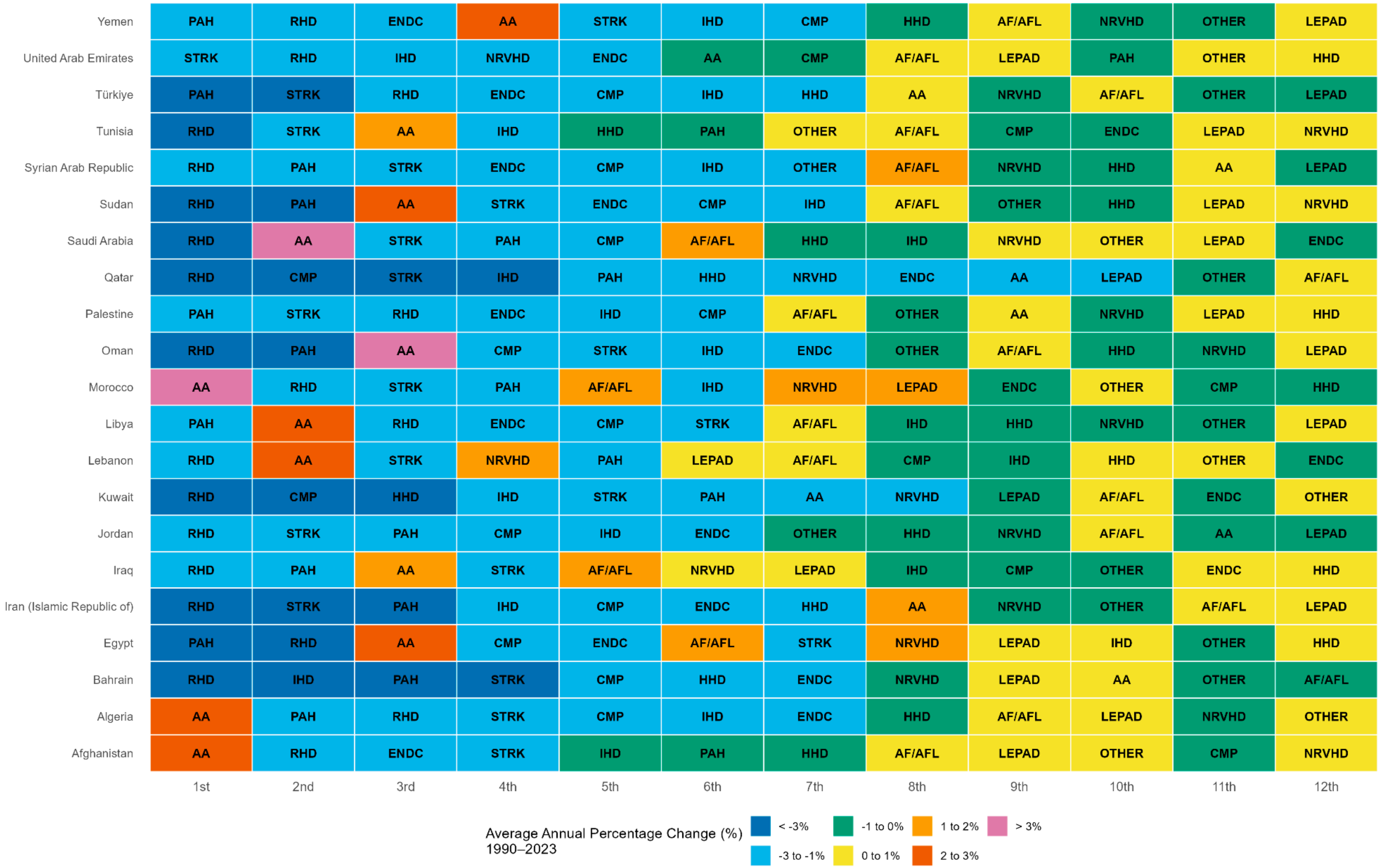

Figure S9. Heatmap of average annual percentage change (AAPC, %) in age-standardised cardiovascular mortality across 21 NAME countries and 12 cardiovascular causes, 1990–2023. AAPC computed by log-linear regression of age-standardised rates over the full period. Cell labels show AAPC (%); red shades indicate increasing mortality, blue shades decreasing mortality. Abbreviations: IHD, ischaemic heart disease; STRK, stroke; HHD, hypertensive heart disease; AF/AFL, atrial fibrillation and flutter; CMP, cardiomyopathy and myocarditis; RHD, rheumatic heart disease; NRVHD, non-rheumatic valvular heart disease; AA, aortic aneurysm; LEPAD, lower extremity peripheral arterial disease; ENDC, endocarditis; PAH, pulmonary arterial hypertension; OTHER, other cardiovascular and circulatory diseases.

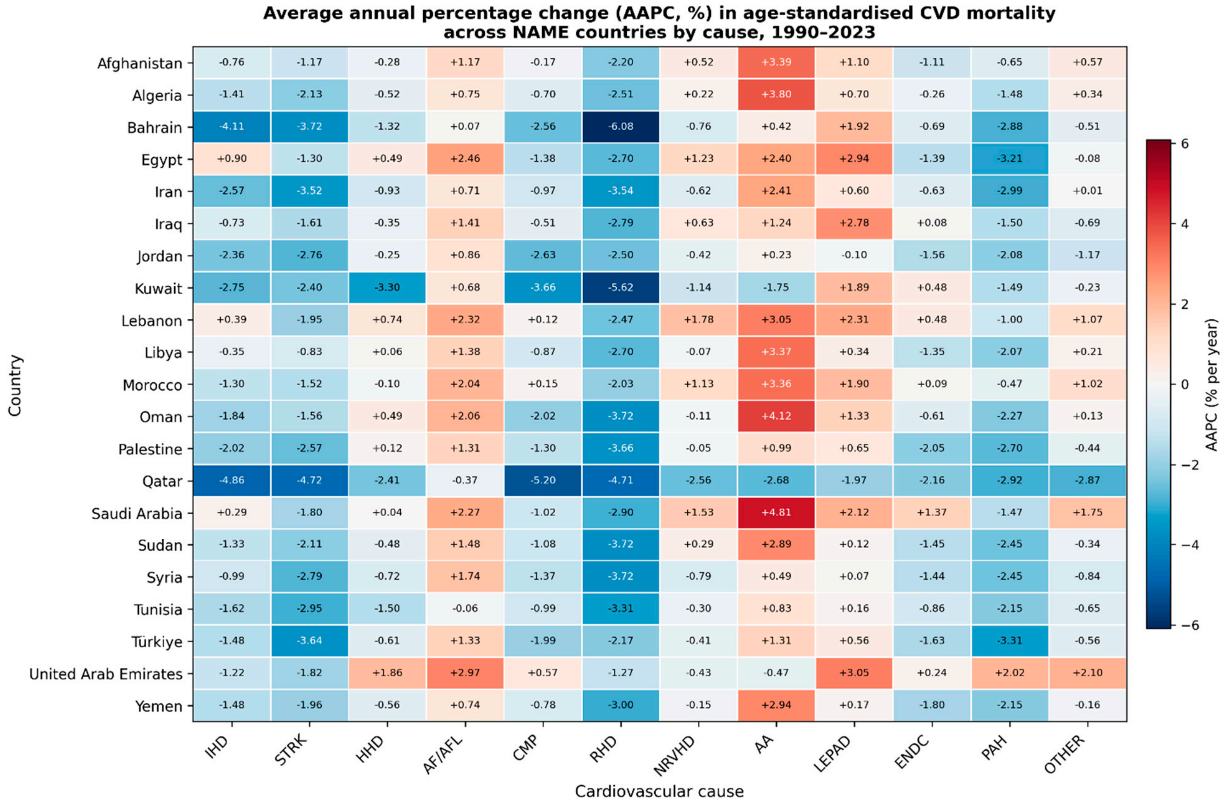

Figure S10. Comparison of NAME countries in terms of ASR of mortality and DALYs due to different CVD causes, 2023. Abbreviations: ASR, age-standardised rate; CVD, cardiovascular disease; DALYs, disability-adjusted life years; NAME, North Africa and Middle East.

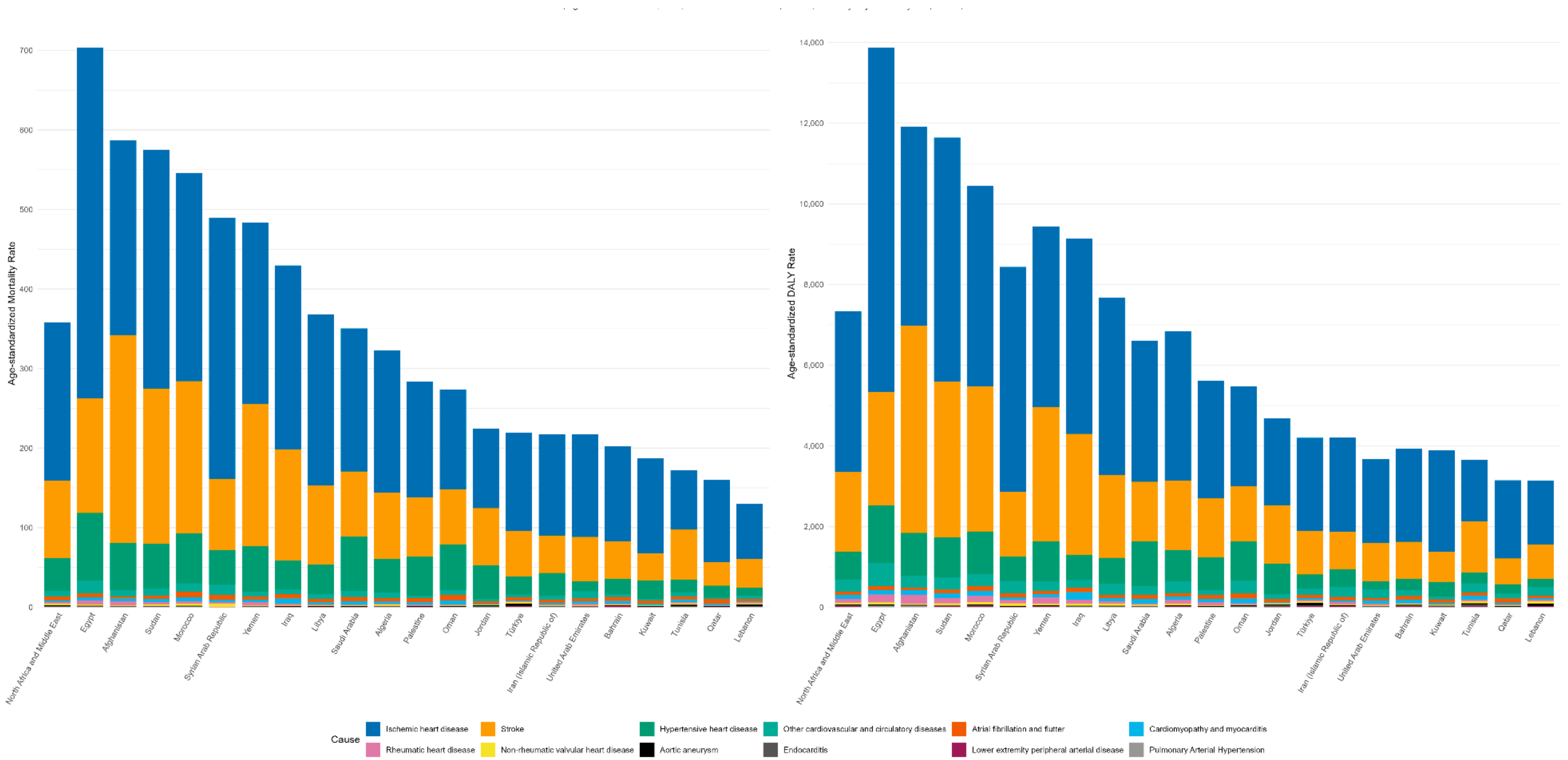

Checklist S1. The RECORD statement – checklist of items, extended from the STROBE statement, that should be reported in observational studies using routinely collected health data.

	Item No.	STROBE items	Location in manuscript where items are reported	RECORD items	Location in manuscript where items are reported
**Title and abstract**					
	1	(a) Indicate the study's design with a commonly used term in the title or the abstract (b) Provide in the abstract an informative and balanced summary of what was done and what was found	Title; Abstract	RECORD 1.1: The type of data used should be specified in the title or abstract. When possible, the name of the databases used should be included.<br><br>RECORD 1.2: If applicable, the geographic region and timeframe within which the study took place should be reported in the title or abstract.<br><br>RECORD 1.3: If linkage between databases was conducted for the study, this should be clearly stated in the title or abstract.	Title; Abstract
**Introduction**					
Background rationale	2	Explain the scientific background and rationale for the investigation being reported	Introduction, paragraphs 1–4		
Objectives	3	State specific objectives, including any prespecified hypotheses	Introduction, last paragraph		
**Methods**					
Study Design	4	Present key elements of study design early in the paper	Materials and Methods, Data		

			Source and Study Design		
Setting	5	Describe the setting, locations, and relevant dates, including periods of recruitment, exposure, follow-up, and data collection	Materials and Methods, Data Source and Study Design		
Participants	6	*(a) Cohort study* - Give the eligibility criteria, and the sources and methods of selection of participants. Describe methods of follow-up<br>*Case-control study* - Give the eligibility criteria, and the sources and methods of case ascertainment and control selection. Give the rationale for the choice of cases and controls<br>*Cross-sectional study* - Give the eligibility criteria, and the sources and methods of selection of participants<br><br>*(b) Cohort study* - For matched studies, give matching criteria and number of exposed and unexposed<br>*Case-control study* - For matched studies, give matching criteria and the number of controls per case	Materials and Methods, Data Source and Study Design	RECORD 6.1: The methods of study population selection (such as codes or algorithms used to identify subjects) should be listed in detail. If this is not possible, an explanation should be provided.<br><br>RECORD 6.2: Any validation studies of the codes or algorithms used to select the population should be referenced. If validation was conducted for this study and not published elsewhere, detailed methods and results should be provided.<br><br>RECORD 6.3: If the study involved linkage of databases, consider use of a flow diagram or other graphical display to demonstrate the data linkage process, including the number of individuals with linked data at each stage.	Methods, pp. 5-6
Variables	7	Clearly define all outcomes, exposures, predictors, potential confounders, and effect modifiers. Give diagnostic criteria, if applicable.	Materials and Methods, Disease Classification and Epidemiological Metrics	RECORD 7.1: A complete list of codes and algorithms used to classify exposures, outcomes, confounders, and effect modifiers should be	Methods, p. 5 (ICD-10 codes for the 12 CVD subtypes listed in

				provided. If these cannot be reported, an explanation should be provided.	Disease Classification)
Data sources/ measurement	8	For each variable of interest, give sources of data and details of methods of assessment (measurement).<br>Describe comparability of assessment methods if there is more than one group	Materials and Methods, Disease Classification and Epidemiological Metrics		
Bias	9	Describe any efforts to address potential sources of bias	Materials and Methods, Disease Classification and Epidemiological Metrics		
Study size	10	Explain how the study size was arrived at	Materials and Methods, Disease Classification and Epidemiological Metrics		
Quantitative variables	11	Explain how quantitative variables were handled in the analyses. If applicable, describe which groupings were chosen, and why	Materials and Methods, Disease Classification and Epidemiological Metrics		
Statistical methods	12	(a) Describe all statistical methods, including those used to control for confounding<br>(b) Describe any methods used to examine subgroups and interactions<br>(c) Explain how missing data were addressed<br>(d) *Cohort study* - If applicable, explain how loss to follow-up was addressed	Materials and Methods, Trend Analysis		

		Case-control study - If applicable, explain how matching of cases and controls was addressed<br>*Cross-sectional study* - If applicable, describe analytical methods taking account of sampling strategy<br>(e) Describe any sensitivity analyses			
Data access and cleaning methods		..	Not applicable	RECORD 12.1: Authors should describe the extent to which the investigators had access to the database population used to create the study population.<br><br>RECORD 12.2: Authors should provide information on the data cleaning methods used in the study.	Methods, pp. 5-6
Linkage		..	Not applicable	RECORD 12.3: State whether the study included person-level, institutional-level, or other data linkage across two or more databases. The methods of linkage and methods of linkage quality evaluation should be provided.	No data linkage was performed; all data were extracted from the GBD Results Tool.
**Results**					
Participants	13	(a) Report the numbers of individuals at each stage of the study (*e.g.*, numbers potentially eligible, examined for eligibility, confirmed eligible, included in the study, completing follow-up, and analysed)	Not applicable	RECORD 13.1: Describe in detail the selection of the persons included in the study (*i.e.,* study population selection) including filtering based on data quality, data availability and linkage. The selection of included persons can be described in the text and/or by means of the study flow diagram.	Methods, p. 5

		(b) Give reasons for non-participation at each stage.<br>(c) Consider use of a flow diagram			
Descriptive data	14	(a) Give characteristics of study participants (*e.g.*, demographic, clinical, social) and information on exposures and potential confounders<br>(b) Indicate the number of participants with missing data for each variable of interest<br>(c) *Cohort study* - summarise follow-up time (*e.g.*, average and total amount)	Results		
Outcome data	15	*Cohort study* - Report numbers of outcome events or summary measures over time<br>*Case-control study* - Report numbers in each exposure category, or summary measures of exposure<br>*Cross-sectional study* - Report numbers of outcome events or summary measures	Results; Table 1		
Main results	16	(a) Give unadjusted estimates and, if applicable, confounder-adjusted estimates and their precision (e.g., 95% confidence interval). Make clear which confounders were adjusted for and why they were included<br>(b) Report category boundaries when continuous variables were categorized	Results, Table 1		

		(c) If relevant, consider translating estimates of relative risk into absolute risk for a meaningful time period			
Other analyses	17	Report other analyses done, e.g., analyses of subgroups and interactions, and sensitivity analyses	Results		
**Discussion**					
Key results	18	Summarise key results with reference to study objectives	Discussion, first paragraph		
Limitations	19	Discuss limitations of the study, taking into account sources of potential bias or imprecision. Discuss both direction and magnitude of any potential bias	Limitations,	RECORD 19.1: Discuss the implications of using data that were not created or collected to answer the specific research question(s). Include discussion of misclassification bias, unmeasured confounding, missing data, and changing eligibility over time, as they pertain to the study being reported.	Limitations, p. 15
Interpretation	20	Give a cautious overall interpretation of results considering objectives, limitations, multiplicity of analyses, results from similar studies, and other relevant evidence	Discussion, Conclusion		
Generalisability	21	Discuss the generalisability (external validity) of the study results	Discussion, last paragraphs		
**Other Information**					
Funding	22	Give the source of funding and the role of the funders for the present study and, if applicable, for the original study on which the present article is based	Declarations: Funding		

Accessibility of protocol, raw data, and programming code		..	Not applicable	RECORD 22.1: Authors should provide information on how to access any supplemental information such as the study protocol, raw data, or programming code.	Declarations: Availability of data and materials; Zenodo repository (https://doi.org/10.5281/zenodo.19909178)

*Reference: Benchimol EI, Smeeth L, Guttmann A, Harron K, Moher D, Petersen I, Sørensen HT, von Elm E, Langan SM, the RECORD Working Committee. The REporting of studies Conducted using Observational Routinely-collected health Data (RECORD) Statement. PLoS Med. 2015;12(10):e1001885.

*Checklist is protected under Creative Commons Attribution (CC BY) license.
